# Data Donation as a Method to Measure Physical Activity in Older Adults: Cross-Sectional Web Survey Assessing Consent Rates, Donation Success, and Bias

**DOI:** 10.2196/69799

**Published:** 2025-09-26

**Authors:** Florian Keusch, Bella Struminskaya, Joris Mulder, Stein Jongerius

**Affiliations:** 1 Department of Sociology School of Social Sciences University of Mannheim Mannheim Germany; 2 Department of Methodology & Statistics Faculty of Social and Behavioural Sciences Utrecht University Utrecht The Netherlands; 3 Centerdata Tilburg University Tilburg The Netherlands

**Keywords:** smartphones, consent, health apps, self-report, data donation

## Abstract

**Background:**

Accurate measurement of physical activity (PA) is key to identifying determinants of health and developing appropriate interventions. Self-reports of PA (eg, in surveys or diary studies) often suffer from measurement error. Providing study participants with wearable devices that passively track PA reduces reactivity and recall error but participants’ noncompliance and high device costs are problematic. Many older adults now have smartphones that track PA. Based on legal requirements, data controllers (eg, health apps) must provide users with access to their data, and individuals can request and donate these data for research. This user-centric approach provides researchers with access to individual-level data, and it gives users control over what data are shared.

**Objective:**

We conduct a first test of the data donation approach for PA data among older adults. We study (1) how willing and successful older adults are to donate their PA data from different smartphone apps, (2) what drives donation of PA data at the different stages of participation, and (3) what biases arise from selective data donation.

**Methods:**

To answer our research questions, we use cross-sectional observational data from a probability-based online panel of the Dutch general population. A total of 2086 members of the Longitudinal Internet Studies for the Social Sciences panel aged 50 years and older completed a web survey in 2024. All iPhone and Android smartphone owners were asked to download passively collected PA data from their devices (Apple Health, Google Location History, or Samsung Health) and donate them via the Port platform.

**Results:**

Out of the 2086 survey participants, 1889 (91%) reported owning an iPhone or Android phone compatible for data donation, 606 (29%) reported willingness to donate PA data, 354 (17%) started the data donation, and 256 (12%) successfully provided a data package. Gender, age, educational attainment, monthly personal net income, smartphone usage behavior, privacy- and trust-related attitudes, and type of health app from which the data were requested correlated with behavior at the different stages of study participation. Self-reported reasons for nonwillingness to donate related mainly to expected technical issues, privacy concerns, and perceived usefulness. Compared with the entire sample, data donors reported better health, fewer health-related limitations, fewer difficulties performing tasks, and more PA.

**Conclusions:**

Our study shows that data donation from smartphones as part of a probability-based web survey of older adults is a feasible alternative for the measurement of PA, especially for iPhone owners younger than 70 years. Limitations relate to nonparticipation which correlates strongly with characteristics of smartphone ownership and comfort with device use. Substantive bias in health and PA outcomes persists for those who donated in comparison with all survey respondents.

## Introduction

Engagement in physical activity (PA) is the foundation of a healthy lifestyle, elevated immune and psychological function, and decreased mortality [1,2], especially for aging populations [3]. While the accurate measurement of PA is key to identifying determinants of health and developing appropriate interventions, reliable and valid measurement of PA is not trivial.

### Traditional Methods of Measuring PA

To measure PA, researchers usually rely on participants’ self-reports [4]. Large population studies often use standardized PA inventories such as the International Physical Activity Questionnaire [5]. In the Netherlands, the Short QUestionnaire to Assess Health enhancing physical activity is commonly used in the assessment of PA levels [6,7]. While these instruments allow for a relatively fine-grained measurement of different types of PA, they are prone to measurement error due to social desirability, forgetting, and wrongful estimation of the frequency and duration of activities [8,9]. To reduce respondent burden and save questionnaire space, sometimes global measures of PA (eg, average daily hours of moderate or vigorous activity and sedentary behavior) are applied. Apart from the loss of information compared with more fine-grained instruments, one major limitation of these global measures is that they can suffer from misclassification (eg, walking the dog not considered PA) [10,11].

As an alternative to survey-based PA measures, researchers instruct study participants to document all behaviors at fine-grained temporal levels in a diary, the so-called day reconstruction method [12]. While this method should, in theory, provide more accurate estimates of PA than retrospective survey measures, people’s tendency to postpone filling out the diary can produce recall error [13]. Diary studies also induce high respondent burden leading to participant fatigue and dropout [14], one of the reasons why they suffer from particularly low response rates [15]. To reduce burden, diary studies are usually restricted to short reference periods (eg, 1 week).

Recent technological advancement in wearable technology has led to an increase in the use of sensor-based measurements of PA. Researchers now often provide study participants with wrist-, waist-, or thigh-worn accelerometers [16-18], or they specifically recruit people who already own consumer-grade wearables such as smartwatches and fitness trackers [19]. The advantage of passively tracking PA via sensors is that it allows for continuous, fine-gained measurement that alleviates reactivity and error due to forgetting leading to higher accuracy compared with self-reports [20]. However, handing out devices to study participants comes with high costs and noncompliance among participants who are not used to wearing an unfamiliar device [21,22]. Relying entirely on samples of people who bring their own tracking devices leads to the underrepresentation of certain subpopulations, such as men, older people, and those with formally lower educational attainment and lower income, as well as minorities [23,24] and an overrepresentation of people with better health status [24].

In this paper, we will introduce the concept of data donation [25] as an alternative for the measurement of PA in older adults. Data donation builds on the legal requirement of data-collecting platforms (such as health apps) and devices (such as fitness trackers) to give their users access to their own data. The approach combines the advantages of objective measuring PA with sensors from a device that is widely used in the general population—the smartphone—with giving study participants full control over what data are shared with the researchers. While originally developed and applied in the field of communication research [26], we will assess its feasibility for the measurement of PA among older adults in an empirical study with a large sample of members of a probability-based online panel in the Netherlands.

### Data Donation as an Alternative for PA Measurement

#### Overview

Data donation describes a data collection method where people voluntarily contribute their retrospective personal data that were originally generated for a different purpose to research [27]. This data collection approach has gained traction through recent legislative changes such as the European Union’s General Data Protection Regulation [28] and the California Consumer Privacy Act [29] that grant data subjects (ie, individual users) the right of access and transportability to a copy of their personal data from a data controller (eg, health apps, social media platforms, etc) in a machine-readable format. In the literature, the term “Data Download Packages” (DDPs) [30] is now commonly used for the copies of data that a data subject can retrieve from a data controller and share with others, including researchers.

Going directly to the users and asking them to retrieve and donate their DDPs from smartphone apps has several advantages over other methods of digital trace data collection. First, data donation is a user-centered approach that allows researchers to circumvent the increasingly restrictive access that data-controlling platforms have put on Application Programming Interfaces, which used to be a convenient resource for researchers to access individual-level data in the past [31-34]. Second, asking for data donation from smartphones, a device widely used in the general population across the globe [35-37], provides access to data from a much broader population than recruiting people who own smartwatches and other wearable devices [19]. Third, implementing the data donation task as part of a survey allows researchers to further link attitudinal data and other information not recorded in the DDP [38]. Finally, the active involvement of the study participants when downloading and donating retrospective data for research gives them control over what data are actually shared with the researcher. Designated data donation platforms such as the Open-Source Data Donation Framework [39] and Port [40] enable researchers to execute scripts that preprocess DDPs (eg, aggregate data and delete all personally identifiable information) locally on a user’s device and give study participants the option to review and curate their data before donation. Data donation is thus considered a more ethical way of collecting personal digital data than other passive data collection methods that continuously collect prospective data in the background of the device [41].

When conceptualizing data donation as a technology, we can use the Technology Acceptance Model (TAM) [42,43] and its extension [44] to explain participation behavior, similar to other studies that use smartphone apps for data collection [45]. According to the TAM, technology adoption is a function of perceived ease of use, perceived usefulness, and perceived trustworthiness of the technology. Data donation requires participants to perform a series of steps: requesting and retrieving their DDPs from a data controller and subsequently donating them for research [46]. Depending on the platform from which the DDPs are downloaded, these tasks might vary in complexity, meaning that researchers will need to provide clear and detailed guidance, especially for less tech-savvy participants [38,47,48]. The potentially rather onerous multistep process of data donation (ease of use) and concerns about the sensitivity of the data (trustworthiness) might reduce the willingness of individuals to donate PA data for research. At the same time, individuals might be motivated by the information they receive as part of the data donation process about their own user behavior or a monetary incentive (usefulness). However, if certain subgroups are more or less likely to donate their data, then systematic nonparticipation error (ie, bias) might arise.

So far, the literature on data donation as a method for digital data collection has focused primarily on applications in communications research with generally younger users of specific social media platforms, including Facebook [49-52], Instagram [47,52,53], and WhatsApp [54,55]. It is well known that digital skills are negatively correlated with age [56-58]. However, the adoption of technology, in particular smartphones, among older age groups has grown markedly recently [59]. It is thus crucial to understand whether data donation is feasible in populations of older adults, what drives the decision to donate data in this population, and what consequences selective nonparticipation has for data quality.

#### Data Donation Participation Rates

From research with users of specific social media platforms, we know that while a relatively large proportion of individuals initially states that they are willing to donate their data, a much smaller proportion usually successfully does so. For example, in a study with 388 adolescent Instagram users in the Netherlands, 287 (74%) obtained parental assent, 148 (38%) gave informed assent, and 102 (26%) eventually uploaded 110 useable Instagram DDPs [47]. In a study with 913 German Facebook users, 725 (79%) reported being willing to donate Facebook data, and eventually 345 (38%) provided at least 1 DDP [49]. Another 2 studies in Germany showed that while 63% and 52% of participants reported an intention to donate data from 1 of 4 social media platforms, only 20% and 12% actually donated their data [60].

So far, few studies have collected PA data using data donation. Toepoel et al [61] asked 1119 Dutch smartphone and fitness tracker owners about their willingness to copy daily PA data from the device into a questionnaire or upload it to a portal. Willingness to upload varied between 33% and 49% depending on whether participants used only the smartphones to monitor PA or had an activity tracker. Kompier et al [62] invited 615 members of the Longitudinal Internet Studies for the Social Sciences (LISS), a probability-based online panel in the Netherlands, to wear an ActivPAL PA tracker for 1 week. A total of 503 (82%) panel members did so of whom 221 (45%) indicated that they also wore their own PA tracker (Apple watch, Samsung watch, Garmin watch, or a Fitbit) during the study period. Those who wore their own devices were asked to manually copy the data from their personal PA tracker for 1 week into a web survey, of whom 155 (221, 70%) did so. Sixty-one individuals owned a Fitbit and were then also asked to provide a DDP from Fitbit as part of the survey, which 34 (15% of those who wore their own device) individuals did. Silber et al [63] asked 2040 German smartphone owners to donate their health app data. Out of 1020 iPhone owners, 374 (37%) consented to donate Apple Health data, and 94 (9%) successfully provided a DDP. Out of 1020 Samsung smartphone owners, 425 (42%) consented to donate Samsung Health data, and 26 (3%) successfully provided a DDP.

In summary, earlier research finds different donation rates ranging from 3% to 49%. These studies differed in a number of characteristics, including the type of data requested and the study population. With our first research question (RQ), we extend the existing research on data donation in 2 directions: first, we specifically study willingness and data donation behavior among older adults, and second, we focus on PA data from health apps on smartphones.

- RQ1: How willing and able are older adults to donate their PA data from different smartphone apps?

#### Predictors of Data Donation at Different Stages

Several studies have shown that different sociodemographic (eg, age, educational attainment, and income), behavioral (eg, frequency of technology and media use), and attitudinal characteristics (eg, privacy concerns and trust in research organizations) predict whether individuals donate their data for research. However, the findings are not always consistent in how these variables influence the donation decision [47,49,63,64]. One possible explanation for this inconsistency in findings is that the global assessment of the data donation outcomes might obscure the mechanisms at play at the individual stages of the process. Consenting to a request to data donation for research and actually going through the process of downloading data and successfully donating them are conceptually separate and independent steps that might be influenced by different factors in line with the TAM. Studies that focused on the first step by asking about the (hypothetical) willingness of people to provide digital personal data for research (either through data donation or other means of data collection) show that concerns about privacy of the data and security of the data-handling process as well as trust in the organization that collects the data (ie, trustworthiness of the technology) play an important role in the initial decision to consent to study participation [45,60,65-67]. This is corroborated by self-reports of people who declined the request to participate in actual data donation studies [49,52]. The success of the second step, that is, donation conditional on consent, is likely influenced by how cumbersome the process is and whether respondents encounter any technical problems [49,60,68] (ie, ease of use of the technology). Thus, factors such as age, education, and familiarity and comfort with technology might be predictive of successful data donation at this stage.

In our study, we expand the existing literature by deliberately separating out the individual steps in the data donation process and studying the factors that influence participation outcome in a more nuanced way.

- RQ2. What drives donation of PA data at different stages of participation?

As part of the investigation into the factors that drive data donation, we are also interested in the effect of showing respondents a stylized example of the data they will be donating as part of the request. Studies on the willingness to share and actual sharing of prospective data from apps and wearables show that participants are more likely to do so if they are promised to have agency over their data, for example, when given the option to view data, revoke consent, receive feedback, and temporarily turn off data collection [45,66,67]. We might find similar effects for donation of retrospective PA data. Seeing an example of what data will be shared with the researcher might also increase the perceived usefulness of the data donation task in line with the TAM. At the same time, showing a visualization of PA data to potential study participants might have an adverse effect: seeing how these data look may trigger privacy concerns among participants that lead them to decide not to donate altogether.

- RQ2a. What effect does showing respondents an example of the type of PA data they will be donating as part of the request have on the data donation behavior?

#### Bias Due to Selective Data Donation Participation

Eventually, low rates of willingness to donate and actual donation are just 1 of 2 components determining whether the results of a study suffer from bias. Another factor to consider is whether the people who successfully donate their data differ systematically from the target population in the variables of interest of a study [69,70]. However, few data donation studies have assessed whether the sample of donors is biased in the variables that should be measured with the donated data. In a study among Facebook users, no systematic differences in self-reported frequency of Facebook use were found between donors and nondonors [49]. Another media and communications study found that politically interested and left-leaning respondents and those with higher social media use were more likely to donate data from social media platforms [60]. Studies in the health domain often suffer from a “healthy volunteer bias,” that is, those who decide to participate in health research tend to be, on average, more health conscious than nonparticipants [71-76]. With our study, we expand the current literature on data donation by exploring not only bias regarding sociodemographics and privacy-related concerns but also substantive PA and health outcomes.

- RQ3. What bias, if any, does arise from selective data donation participation?

## Methods

### Web Survey

To answer our RQs, we implemented PA data donation in a web survey among members of the LISS panel. The LISS panel is a probability-based online panel in the Netherlands that administers monthly web surveys to respondents who were recruited through drawing an initial random sample from the Dutch population register in 2007 [77]. Since the original recruitment, the LISS panel has been refreshed several times by drawing stratified and random samples of the Dutch population [78]. In January and March of 2024, 2345 members of the LISS panel aged 50 years or older were invited to participate in the web survey. The questionnaire contained questions about sociodemographics, self-rated health, chronic illness, health-related difficulties and limitations, PA in the past 7 days, general privacy concerns, perceived privacy of different types of data, trust in various institutions, and smartphone ownership and use (see Multimedia Appendix 1 for the full questionnaire). Respondents who indicated that they own an iPhone or an Android smartphone were asked at the end of the questionnaire whether they would be willing to donate PA data from their smartphones.

Out of the 2345 invited LISS panel members 2094 (89.3%) started the survey. A total of 2086 (89.9%) members completed the questionnaire up until at least the section on smartphone ownership and use right before the data donation section—the analysis sample for our study (see Multimedia Appendix 2 for descriptive statistics of the sample). To test whether our analysis sample differs from the gross sample invited to the survey, we conducted additional analyses using data from the LISS panel sociodemographic profile data. We find small effects only for age and employment status and correcting for them using weights in our analytical models does not change the substantive conclusions we draw from the findings (Multimedia Appendix 3).

### Data Donation

Respondents were asked to donate PA data from 1 of 3 apps, depending on their smartphone. Respondents who reported owning an iPhone were asked to donate daily step count data from the Apple Health app. Android smartphone owners who had indicated owning a Samsung smartphone and using the Samsung Health app were asked to donate daily step count data from the Samsung Health app. All remaining Android smartphone respondents were asked to donate daily traveled distance data from Google Location History. Note that while Google Location History also includes information on tracks, locations, and places [76], we requested information only on the daily distance traveled in kilometers. Depending on how long respondents had used an app, data from up to 2017 until the day of the request made to the data controller (ie, Apple, Samsung, or Google) could be donated.

**Figure 1:**
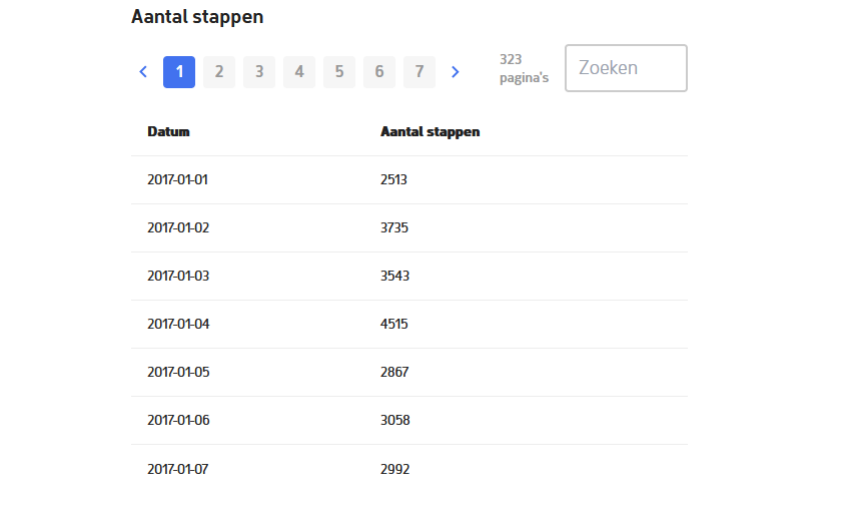
Screenshot of stylized example of physical activity data shown to random half of respondents.

As part of the introduction to the data donation task, we implemented an experiment varying whether or not respondents saw an example screenshot of the PA data they would donate (RQ2a; Figure 1). The screenshot shows a stylized example of a dummy dataset with dates and number of steps per day (for Apple Health and Samsung Health) or daily distance traveled (for Google Location History). The image shown in the experiment has the exact same design as the last screen that study participants would see right before donating their data. For all 3 app conditions, one random half of the respondents received the data donation introduction with and the other half without the screenshot.

Those who were not willing to donate were asked for their reason for refusal in an open-ended question. Those who agreed to donate their PA data were guided through the data donation process via multiple pages including screenshots and detailed instructions in the web survey. Depending on the app from which the data were to be downloaded, the description included 9 (Apple Health), 11 (Google Location History), or 17 (Samsung) steps. After walking respondents through the data donation process, respondents were asked whether they were able to successfully request the DDP from the data controller and store it on their phone (for Apple and Samsung). For Google, respondents needed to wait to receive an email that the data were ready until they could continue with the questionnaire. The LISS panel sent up to 2 reminders to panel members who had not completed all parts of the study.

Those who had successfully accessed a DDP received a URL and a QR code that sent them to the data donation portal. The data donation was implemented on the Port platform [40] where the DDP was locally processed on the users’ device, meaning that the data did not leave respondents’ device at this stage. At this point, the DDP was stripped of all personally identifiable information and data were aggregated to daily step counts (Apple Health and Samsung Health) or daily distance traveled in kilometers (Google Location History). Before the actual data donation step, that is, before uploading the data extracted from the DDP to the server, respondents were shown a table with all the information that would be shared with the researchers, and respondents were allowed to delete specific entries. To finally send the data to the researchers, respondents needed to click “donate” or they could abandon the data donation at this stage. After the data donation, respondents were automatically rerouted to the web survey, and they were asked whether they were successful in donating the data, whether they experienced any problems when trying to donate the data, and if so, what kind of problems.

### Ethical Considerations

All procedures of the study were approved by the ethics committee of the Faculty of Social and Behavioural Sciences of Utrecht University (23-0494). Informed consent was obtained twice: first, when participants joined the LISS panel (covering panel participation and data use), and again at the start of the web survey (covering this specific study). Participants could opt out of the study at any point. PA data from smartphones were collected only from those participants who requested and downloaded their data from the platform and then donated their DDPs. In a preprocessing step locally executed on the participants device, DDPs were stripped of all personally identifiable information and only aggregated data (daily step counts or daily distance traveled in kilometers) could be donated. Anonymized data for this project are available in the LISS Data Archive [79]. Respondents received an equivalent of €15 per hour for completing the survey. As an additional incentive, respondents received €5 for a successfully donated data package. On January 1, 2024, €1 was valued at US $1.08.

### Statistical Analysis

All analysis was conducted using R (version 4.5.1; R Core Team) [80], and all analysis code can be found on the web [81]. To answer RQ1 about participation rates in the data donation study, we calculate the share of respondents who (1) reported owning an iPhone or an Android smartphone necessary to participate in the data donation study, (2) reported being willing to share their PA data, (3) started the data donation process on the Port platform, and (4) successfully provided data.

To answer RQ2 about the predictors of participation, we estimate 4 logistic regression models, 1 for each of the 4 outcomes from RQ1 as the dependent variable. We use five sets of predictors in our models: (1) sociodemographic characteristics (gender, age, educational attainment, employment status, monthly personal net income, household size, and urbanicity), (2) privacy- and trust-related measures (general privacy concern and 3 indexes for perceived privacy of personal information, trust in government and research institutions, and trust in technology companies), (3) health measures (self-rated health, chronic illness, classification based on BMI [82], and indexes for health-related limitations in 3 domains and for difficulties performing 10 different tasks in everyday life), and (4) PA-related variables (number of days with strenuous and moderate PA in the past 7 days; number of days with walking, biking, and running in the past 7 days; number of hours of sedentary behavior per day in the past 7 days; and dummy for having spent time outside the home yesterday). Except for model 1 (predicting ownership of a smartphone) we also included (5) number of smartphone activities and type of data requested as predictors. To answer RQ2a about the effect of showing an example of the data on donation behavior, we added a dummy variable to models 2-4 that indicated whether or not a respondent was shown an example of data before the willingness question.

For some categorical variables, we collapsed categories due to small cell counts. Multimedia Appendix 2 provides an extensive overview of all original and recoded variables and the creation of indexes. We used casewise deletion for missing values in our models. For ease of interpretation, we calculated average marginal effects (AMEs) using the margins package in R [83], and we report AMEs and their 95% CIs to quantify the relationship between the independent and the dependent variables in our models. Given the large number of predictors in our models, we also specify more parsimonious models that include only the sociodemographic variables together with all the variables from 1 of the 4 other thematic blocks (privacy and trust, health, PA, and smartphone-related variables). The results of these models (Multimedia Appendix 4) show that our findings are robust, and we thus interpret the results of the full models here.

Based on the answers to an open-ended question asked to people who were not willing to donate their PA data, we further analyze reasons for refusal to participate in the study. We code the answers using an updated coding scheme developed for an earlier data donation study [49].

To study bias due to selective nonparticipation (RQ3), we compare the full sample including all respondents to the web survey with the individuals who provided PA data on all variables that were used as predictors in models 1-4. To facilitate interpretation of bias, we created terciles (low, medium, and high) for all noncategorical variables (ie, all index measures, number of days respondents had performed different PAs, and number of sedentary hours; Multimedia Appendix 2) to show potential overrepresentation and underrepresentation among donors in these groups. Following earlier work [84-86], we calculate bias in a variable of interest (*y*) as the difference in proportions between the full sample (f) and the donors (d) as



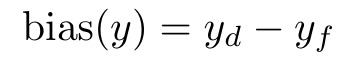



The SE of these differences is calculated as



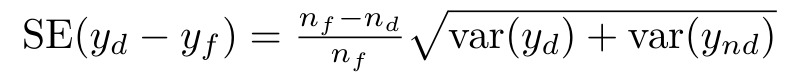



where nd denotes nondonors.

The results of the multivariate models provide information about which variables predict participation when controlling for various factors, allowing for theoretical exploration of participation decisions (RQ2). The bias measures will demonstrate from a practical perspective what systematic errors arise in these variables, in particular, the ones that ought to be measured with the donated data, given selective participation.

## Results

### Data Donation Participation Rate

As shown in Table 1, out of the 2086 respondents in the survey, 1889 (91%) reported owning an iPhone or an Android smartphone necessary for PA data collection. Between the question about smartphone ownership and the willingness to donate data question, 6 participants dropped out of the survey, leaving 1883 who were asked to donate PA data. About one-third of these respondents (606, 29% of the full survey sample) indicated willingness to donate data and were then guided through the data donation process. A total of 354 respondents (17% of the full survey sample or 58% of the willing sample) started the data donation process on the Port platform. About three-quarters of respondents who started the donation process successfully donated data (256, 12% of the full survey sample).

**Table 1:** Data donation participation rate.

Sample	Values, n (%)
Full sample	2086 (100)
Owns iPhone or Android phone	1889 (91)
Willingness question asked	1883 (90)
Willing to donate data	606 (29)
Started donation	354 (17)
DDP^a^ received	256 (12)

^a^DDP: Data Download Package.

### Predictors of Data Donation at Different Stages

Four logistic regression models predict participation behavior in the various stages of data donation conditional on the previous stage: owning an iPhone or an Android smartphone (model 1), willing to donate data conditional on owning an iPhone or an Android smartphone (model 2), starting data donation conditional on willingness (model 3), and successfully donating data conditional on starting data donation (model 4). The main results of the 4 models are summarized in Textbox 1. The full regression results for all 4 models can be found in Multimedia Appendix 5.

Model 1 (Table S1 in Multimedia Appendix 5) shows that for our sample of adults aged 50 years and older in the Netherlands, the predicted probabilities of owning an iPhone or an Android smartphone among men are 5 percentage points (PP) lower than those among women (AME –0.05, 95% CI –0.08 to –0.02). Device ownership decreases significantly with age; compared with the youngest age group in our sample (aged 50-54 years), the predicted probabilities of owning an iPhone or an Android smartphone are 8 PP (AME –0.08, 95% CI –0.14 to –0.03) and 19 PP (AME –0.19, 95% CI –0.27 to –0.12) lower for the 2 oldest groups, those aged 75-79 years and 80 years and older, respectively. While household size, urbanicity, and employment status are not significantly associated with owning the necessary device, ownership significantly increases with educational attainment and monthly personal net income. For example, the predicted probabilities of owning the device among the highest income group (more than €3000 per month) are 11 PP (AME 0.11, 95% CI 0.05-0.17) higher than those among the lowest income group (up to €1000 per month). For people with high educational attainment, the predicted probabilities are 6 PP (AME 0.06, 95% CI 0.03-0.10) higher compared with people with low educational attainment. Interestingly, we find that people who say that they are not very concerned (AME 0.06, 95% CI 0.01-0.11) and those who say they are a little concerned (AME 0.08, 95% CI 0.03-0.14) about their privacy in general have a significantly higher likelihood of owning an iPhone or an Android smartphone than those who are not at all concerned. We find very little relationship between owning an iPhone or an Android smartphone and health-related and PA-related measures. One exception is that people who score higher on the index based on difficulties with 10 tasks have significantly lower predicted probabilities of owning a smartphone; with every additional point on the index, the predicted probability decreases by 4 PP (AME –0.04, 95% CI –0.07 to –0.02).

**Figure 2:**
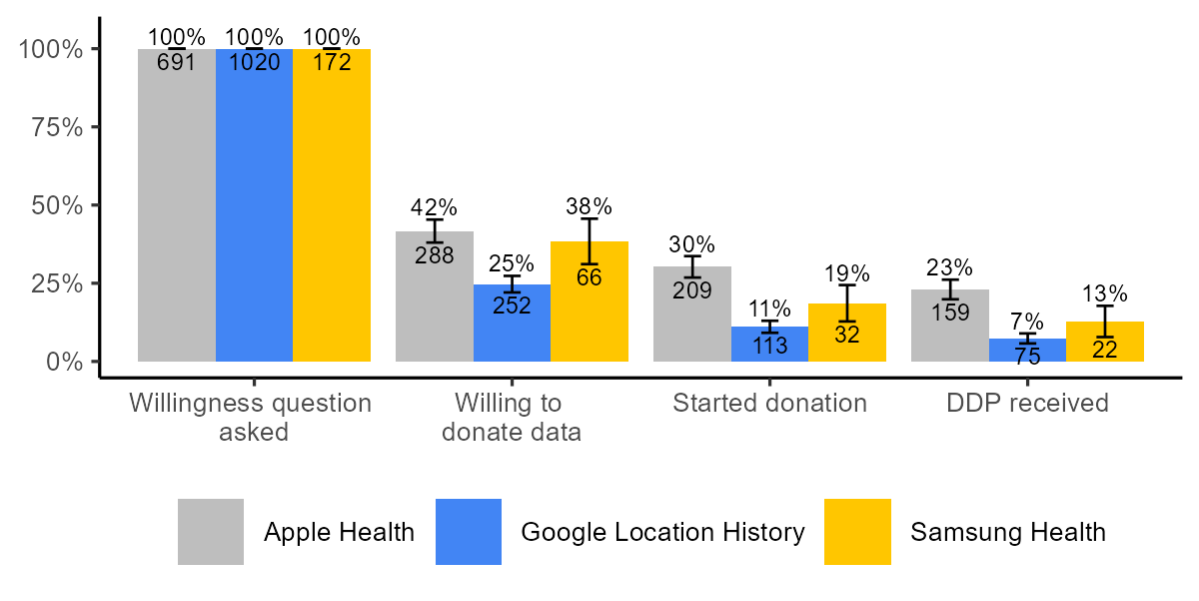
Data donation participation rates by type of data requested. Error bars denote 95% CIs. DDP: Data Download Package.

Based on model 2 (Table S2 in Multimedia Appendix 5), we find that men are significantly more likely to be willing to donate their PA data than women (AME 0.07, 95% CI 0.02-0.11). Likelihood to positively respond to the data donation request significantly decreases with age (75-79 years: AME –0.14, 95% CI –0.24 to –0.03; 80 years and older: AME –0.14, 95% CI –0.26 to –0.02), household size (2-person household: AME –0.07, 95% CI –0.12 to –0.01; 3- and more person household: AME –0.10, 95% CI –0.16 to –0.03), and degree of urbanicity (moderately urban: AME –0.08, 95% CI –0.15 to –0.00; strongly urban: AME –0.11, 95% CI –0.18 to –0.05; and very strongly urban: AME –0.10, 95% CI –0.18 to –0.03). Compared with people with low educational attainment, the predicted probabilities of reporting willingness to donate are 10 PP higher for people with high educational attainment (AME 0.10, 95% CI 0.04-0.16). Willingness to donate decreases with higher values on the index for perceived privacy of personal information (AME –0.04, 95% CI –0.06 to –0.02). Higher scores on the trust toward government and research organizations index are associated with more willingness to donate (AME 0.05, 95% CI 0.01-0.09). We find significantly higher likelihood of willingness among people who report chronic illness (AME 0.06, 95% CI 0.01-0.11), but none of the other health-related or PA-related measures showed a significant correlation with willingness to donate data in the multivariate model. With every additional activity someone reports using their smartphone, the predicted probabilities of being willing to donate increase by 3 PP (AME 0.03, 95% CI 0.02-0.04). Compared with iPhone owners who were asked to donate Apple Health data, the predicted probabilities of being willing to donate data from Google Location History among Android smartphone owners are 14 PP lower (AME –0.14, 95% CI –0.18 to –0.09). This difference is also visible in Figure 2. Interestingly, respondents who were shown a stylized example of how the donated PA data would look like were significantly less likely to be willing to donate (AME –0.05, 95% CI –0.09 to –0.01).

**Significant predictors of participation behavior.**

Model 1: Owning an iPhone or an Android phone

- Female
- Younger age
- Higher monthly personal net income
- Higher educational attainment
- Medium general privacy concern
- More difficulties with tasks

Model 2: Willingness to donate physical activity data conditional on being asked

- Male
- Younger age
- Single-person household
- Less urban
- Higher educational attainment
- Lower perceived privacy of personal information
- Higher trust in government and research institutions
- Chronic illness
- More smartphone activities
- Request for Apple Health data versus Google Location History data
- Not seeing data example

Model 3: Starting to donate physical activity data conditional on willingness

- Younger age
- Higher monthly personal net income
- Higher trust in research and government organizations
- Lower trust in technology companies
- Not being overweight
- Fewer limitations by health in activities
- More smartphone activities
- Request for Apple Health data

Model 4: Successful data donation conditional on starting donation process

- Younger age

A total of 1252 survey respondents who were not willing to donate their PA data answered an open-ended question on the reason for their refusal to donate. Table 2 shows that 27% (339/1252) of the responses pertained to anticipated difficulties with the data donation process, followed by the users’ wish to protect their privacy (269/1252, 21%) and doubts about the usefulness of the data donation for research (257/1252, 20%; see Table S3 in Multimedia Appendix 5 for coded subcategories).

Model 3 (Table S4 in Multimedia Appendix 5) shows that among those who were willing to donate PA data, the likelihood to start the donation process significantly and sharply decreased after age 65 years (65-69 years: AME –0.20, 95% CI –0.34 to –0.06; 70-74 years: AME –0.19, 95% CI –0.35 to –0.04; 75-80 years: AME –0.41, 95% CI –0.59 to –0.24; and 80 years and older: AME –0.44, 95% CI –0.65 to –0.23). We also see that the likelihood to start the data donation process increases with personal monthly net income, with people earning €2501 to €3000 having predicted probabilities that are 18 PP higher (AME 0.18, 95% CI 0.00-0.35) compared with people in the lowest income group (up to €1000). None of the other sociodemographic characteristics show a significant association with the likelihood of starting the data donation process. Trust in government and research organizations is significantly positively associated with starting the data donation process (AME 0.17, 95% CI 0.10-0.23) while trust in technology companies is negatively associated (AME –0.13, 95% CI –0.19 to –0.07). Again, few of the health-related and PA-related predictors show a significant correlation with starting the data donation conditional on willingness. People with a BMI that would categorize them as overweight have a significantly lower likelihood of starting the data donation process (AME –0.09, 95% CI –0.17 to –0.01) than people with a BMI indicating underweight or healthy weight. Lower scores on the index for health-related limitations are associated with higher likelihood to start the data donation (AME –0.05, 95% CI –0.11 to –0.00). The more activities someone reports to do on their smartphone, the higher the likelihood of starting the data donation (AME 0.02, 95% CI 0.00-0.03). Android smartphone owners who were willing to donate PA data from Google Location History or from Samsung Health have predicted probabilities of starting the data donation process that are 24 PP (AME –0.24, 95% CI –0.32 to –0.17) and 22 PP (AME –0.22, 95% CI –0.34 to –0.10) lower, respectively, compared with iPhone owners who were willing to donate Apple Health data (Figure 2).

**Table 2:** Coded answers to open-ended question on reasons for not being willing to donate physical activity data (N=1263).

Code	Reason	Values, n (%)^a^
1	Need to protect privacy	269 (21.3)
2	Concern about data misuse or data breach	44 (3.5)
3	Anticipating difficulties with data donation	339 (26.8)
4	Perceived usefulness of data or research	257 (20.3)
5	Personal reasons	106 (8.4)
6	Other reasons	96 (7.6)
7	Unconcrete refusal	238 (18.8)
8	No reasons provided	100 (7.9)

^a^Each answer could be coded in multiple categories; thus, percentages can add up to more than 100.

Finally, model 4 (Table S5 in Multimedia Appendix 5) predicts successful data donation conditional on starting the data donation process. The results again indicate a decrease in the likelihood of donating data by age. The predicted probability for people aged 70-74 years (AME –0.30, 95% CI –0.55 to –0.06) and 75-79 years (AME –0.38, 95% CI –0.72 to –0.05) is significantly lower than that for the youngest reference group (aged 50-54 years). Neither of the other sociodemographic nor any of the privacy-related, trust-related, and smartphone-related variables are significantly correlated with successful donation at the 5% level. Similarly, successfully completing the data donation is not significantly associated with any of the health-related and PA-related measures in our model.

### Bias Due to Selective Data Donation Participation

The analysis of predictors of participation in our multivariate models sheds light on the correlates of the participation behavior at the individual stages of the data donation process. From a practical perspective, it is also important to understand how the attrition of different subgroups at the individual stages adds up to a systematic deviation in characteristics between those who donated their data and those who did not, that is, bias. Thus, we next move to comparing the full survey sample to those who successfully donated their PA data. We find bias in several sociodemographic, privacy-related, trust-related, smartphone-related, health-related, and PA-related measures (see Table S6 in Multimedia Appendix 5 for detailed data).

**Figure 3:**
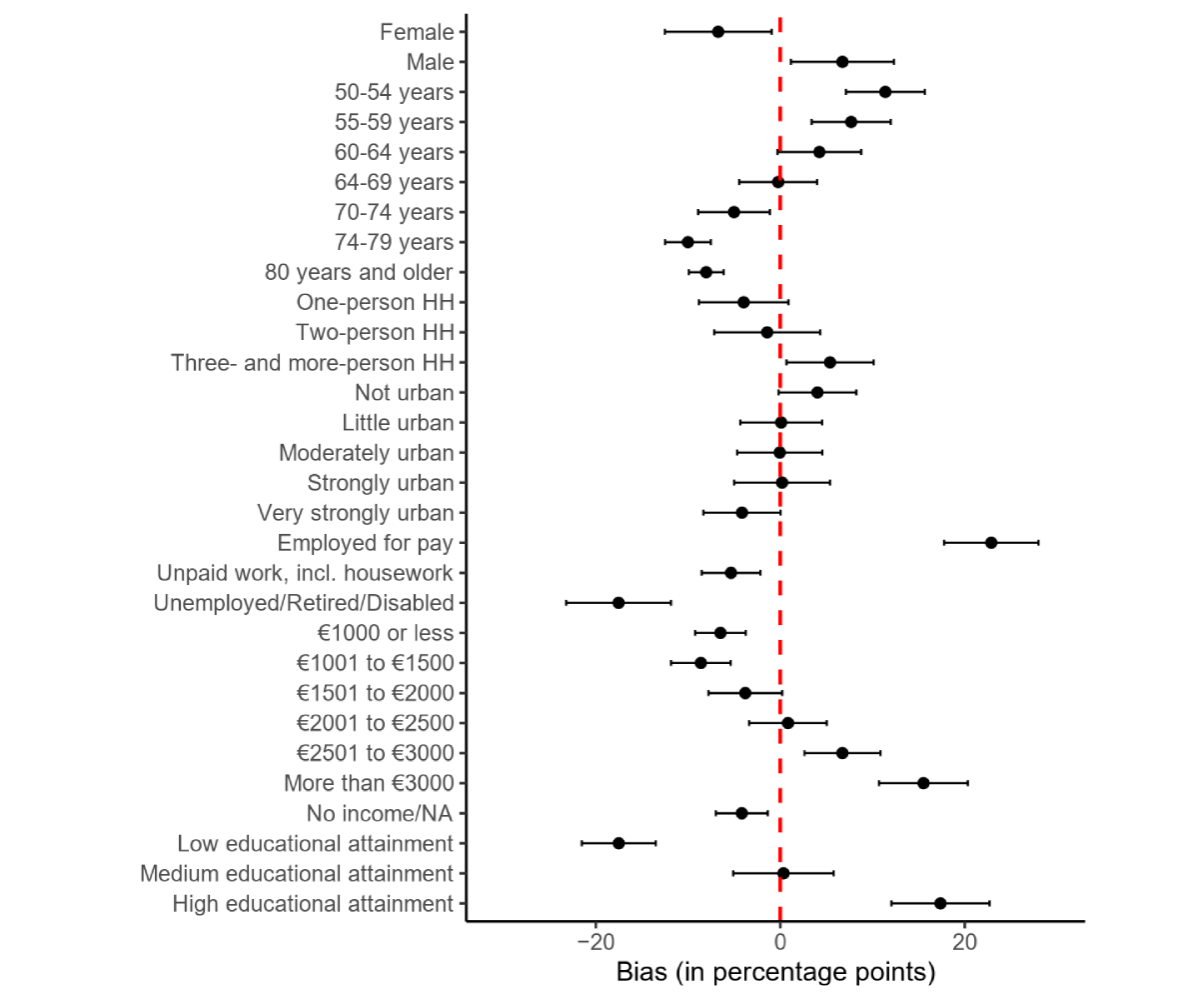
Bias in sociodemographic measures between donors and the full sample. Dots indicate bias point estimators, and bars indicate 95% CIs. HH: household; NA: not available.

In terms of sociodemographics (Figure 3), men are overrepresented among donors by almost 7 PP (95% CI 1.2-12.2), while women are underrepresented. We also see a strong positive bias for the 2 younger age groups (50-54 years: +11.4 PP, 95% CI 7.1-15.7; 55-59 years: +7.7 PP, 95% CI 3.4-12.0) and a negative bias for the 3 oldest age groups (70-74 years: –5.0 PP, 95% CI –8.9 to –1.1; 75-80 years: –10.0 PP, 95% CI –12.5 to –7.5; and 80 years and older: –8.0 PP, 95% CI –9.9 to –6.1). Donors also overrepresent people who are employed for pay by almost 23 PP (+22.9 PP, 95% CI 17.8-28.0), people with high educational attainment by 17 PP (+17.4 PP, 95% 12.0-22.7), and people who live in households with 3 or more people (+5.4 PP, 95% CI 0.7-10.1). At the same time, the group of donors underrepresents those in unpaid work (–5.3 PP, 95% CI –8.5 to –2.2), those unemployed, retired, or disabled (–17.5 PP, 95% CI –23.2 to –11.8), and those with low educational attainment (–17.5 PP, 95% CI –21.5 to –13.5). We find a similar trend for personal monthly net income; those in low-income groups are underrepresented by up to 9 PP (no income/not available: –4.2 PP, 95% CI –7.0 to –1.4; up to €1000: –6.5 PP, 95% CI –9.2 to –3.7; and €1001 to €1500: –8.6 PP, 95% CI –11.8 to –5.4) and those in higher income groups are overrepresented by up to 15 PP (€2501 to €3000: +6.7 PP, 95% CI 2.6-10.9; more than €3000: +15.5 PP, 95% CI 10.7-20.3). We find no significant bias in urbanicity.

**Figure 4:**
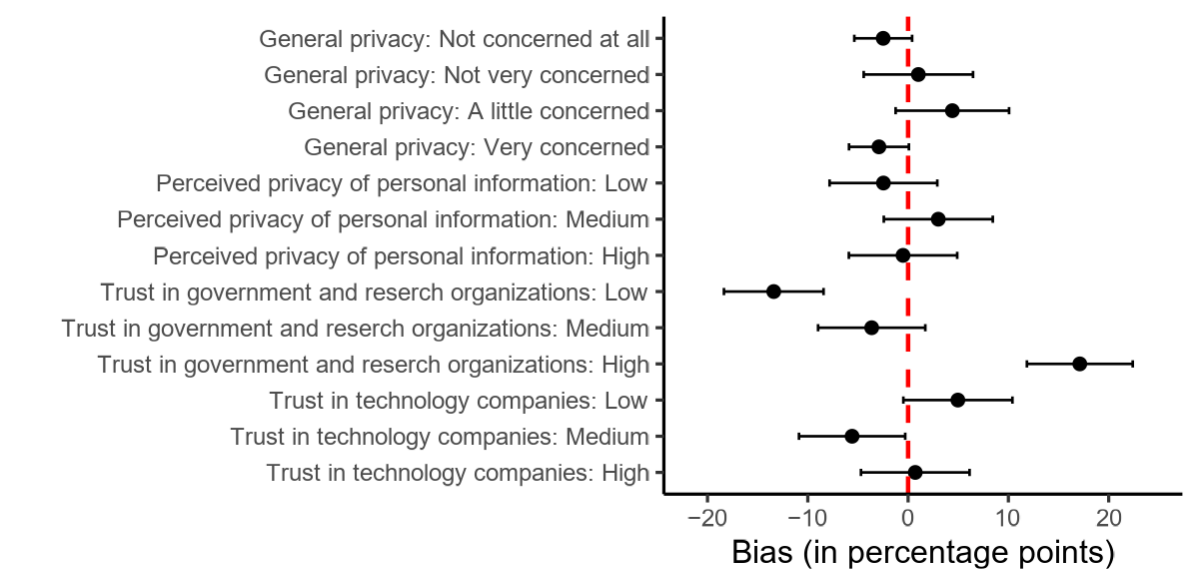
Bias in privacy-related measures between donors and the full sample. Dots indicate bias point estimators, and bars indicate 95% CIs.

As shown in Figure 4, there is very little consistent bias in the measures related to privacy. Compared with the entire sample, people who score in the highest tertile of the trust toward government and research organizations index are overrepresented (+17.1 PP, 95% CI 11.8-22.4) and people who score in the lowest tertile are underrepresented (–13.4 PP, 95% CI –18.4 to –8.4). The bias estimates for the trust measures on technology companies have CIs that include 0.

**Figure 5:**
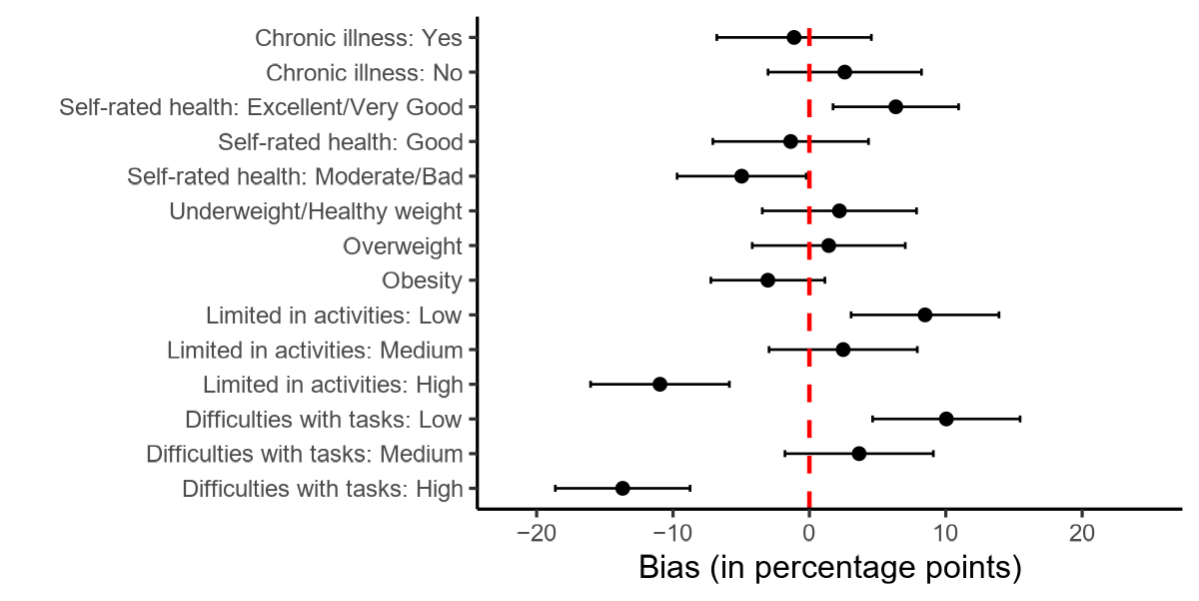
Bias in health-related measures between donors and the full sample. Dots indicate bias point estimators, and bars indicate 95% CIs.

As shown in Figure 5, donors are overrepresenting people who report excellent or very good health by 6.3 PP (95% CI 1.7-10.9) and underrepresenting people reporting moderate or bad health by 5.0 PP (95% CI –9.7 to –0.2). We find more people who report low limits by their health in activities (+8.5 PP, 95% CI 3.1-13.9) and low difficulties with different tasks (+10.0 PP, 95% CI 4.6-15.4) among donors compared with the entire sample. People who fall into the highest groups of health-related activity limits (–11.0 PP, 95% CI –16.0 to –5.9) and difficulties performing tasks due to health issues (–13.7 PP, 95% CI –18.6 to –8.7) are underrepresented among donors. We find no bias in BMI and chronic illness.

**Figure 6:**
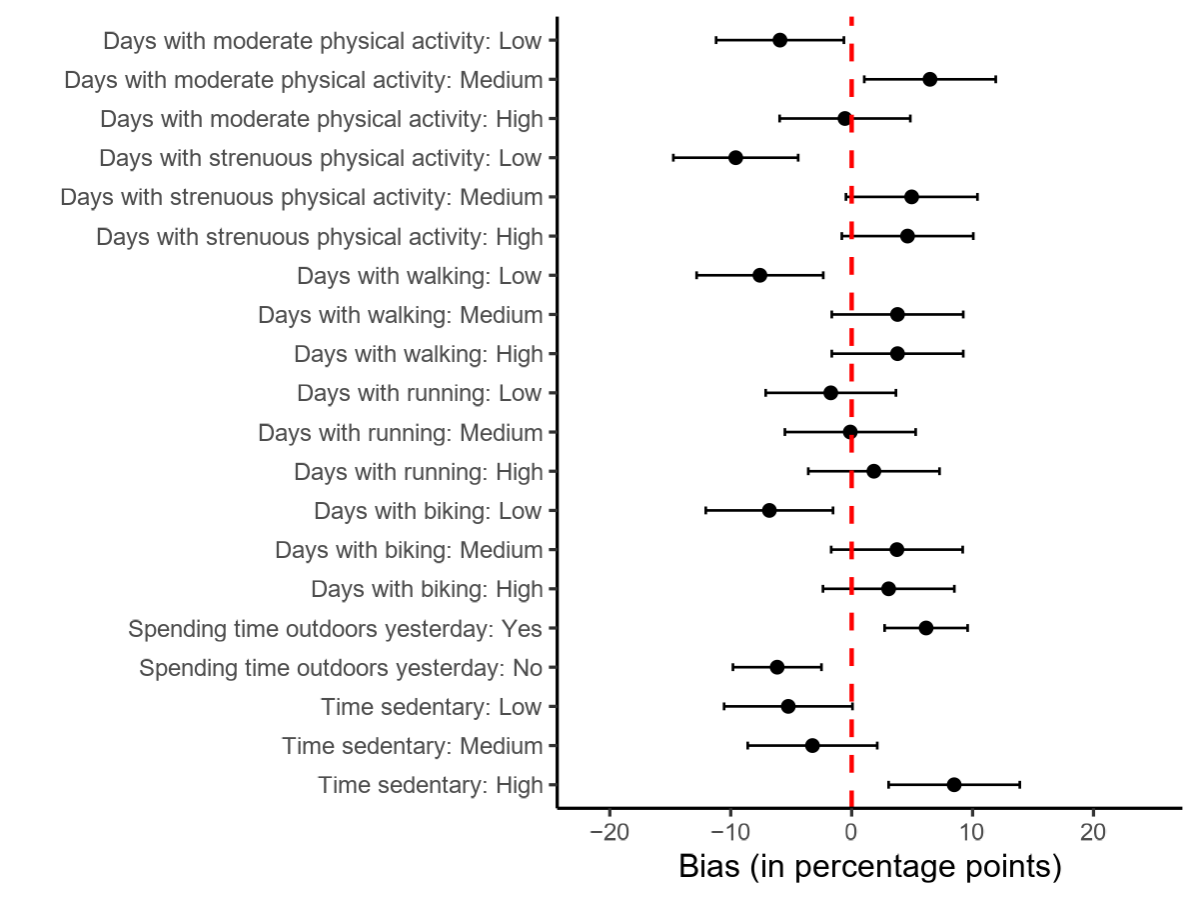
Bias in physical activity–related measures between donors and the full sample. Dots indicate bias point estimators, and bars indicate 95% CIs.

Figure 6 shows that when asked about PA over the course of the past 7 days, fewer donors than people in the entire sample report a number in the lowest tertile for days with moderate PA (–5.9 PP, 95% CI –11.2 to –0.6), strenuous PA (–9.6 PP, 95% CI –14.7 to –4.4), walking (–7.6 PP, 95% CI –12.8 to –2.3), and biking (–6.8 PP, 95% CI –12.1 to –1.5). At the same time, donors overrepresent people in the medium tertile of number of days with moderate PA (+6.5 PP, 95% CI 1.0-11.9) and, interestingly, the highest tertile of hours with sedentary time (+8.5 PP, 95% CI 3.1-13.9). Donors are also overproportionally reporting to have spent some time outdoors yesterday (+6.2 PP, 95% CI 2.7-9.6). The estimates for days with running are not biased.

## Discussion

### Principal Results

We have shown that donation of PA data from smartphones as part of a probability-based online panel is feasible for older adults with certain limitations. In particular, PA data donation works well for iPhone owners younger than 70 years when requesting Apple Health data. Overall, the rates of donation differ substantially between 7% and 23%, depending on what digital platform the data were requested from: Apple Health works much better than Google Location History and Samsung Health. The donation rate for Apple Health in our study is as high, if not higher, compared with studies that have asked people in the general population to download apps that continuously track their activity in the background of the phone [85,87,88]. The large range in participation outcomes between Apple and the other 2 providers might be a reflection of differences in how many steps are necessary and how much time it takes on the individual platforms to request and download PA data. Nonparticipation in data donation also correlates strongly with characteristics related to smartphone ownership and comfort with device use. At all stages of data donation, we observe age effects within the age group of people aged 50 years and older: the older an individual, the less likely to own a smartphone, the less willing to donate, and the less likely to start and to successfully complete the donation process. In sum, these effects lead to stark differences in overall donation rates by age groups. While around 36% of 50- to 54-year-old iPhone owners donated their Apple Health data, the share of people who donated Apple Health data drops to around 17% for people aged 70-74 years and to a mere 3% for people aged 80 years and older. Educational attainment and personal monthly net income are also positively correlated with the likelihood of donating PA data.

Controlling for sociodemographic characteristics, we still see that experience with technology (measured as the number of different smartphone activities users report) is positively correlated with being willing to and starting the donation. Those who are not willing to participate often say that they anticipate technical problems or find the task too difficult. These findings are in line with the idea of perceived ease of technology use from the TAM [42-44]. The more private older adults perceive their PA data to be, the less willing they are to donate, and privacy concerns are the second most named reason for not donating. Again, these findings align with the notion of trustworthiness in the technology postulated by the TAM. Providing a visualization of how the extracted data would look reduces willingness to donate. This finding differs from previous research where showing respondents at the stage of asking for consent how the donated data about their transportation behavior from Google Location History would look like had no effect on donation [89]. Whether this difference is driven by the type of data requested (PA vs mobility), the target population (older adults vs general population), or something else should be the concern of future research.

From a theoretical perspective, it is noteworthy to highlight that the TAM seems to be particularly useful in this age group to explain participation in PA data donation. Reasons related to perceived ease of use, trustworthiness, and usefulness of the data donation all came up prominently in responses to an open-ended question on why people were not willing to donate their PA data. Interestingly, a recent qualitative study with a younger sample did not detect perceived ease of use as one of the main explanations of successful requests to donate data [90]. We could not find that highlighting the usefulness of data donation by showing potential participants a stylized example of the data to be donated would increase participation, quite the opposite. Thus, future research should further test TAM assumptions in different age groups and populations.

Beyond differences in sociodemographics and other covariates, we found that selectivity in participation in the data donation produces substantive bias in health and PA outcomes. Compared with the entire sample of older adults, people who donated their smartphone-based PA data report fewer health-related limitations, less difficulty in performing various tasks, higher levels of PA behavior, and better self-reported health. This finding indicates that while smartphone sensor–based PA measures can potentially improve measurement quality compared with self-report, a “healthy donor bias” exists. Our next step is thus to evaluate the quality of the donated PA data compared with and in combination with self-reports to predict health outcomes. Given the limitations of both data sources (ie, donated sensor–based PA data and self-reports), we currently recommend to integrate data from multiple sources where one source “borrows strength” from the other to address both issues of representation [91] and measurement [92].

From a study design perspective, those who wish to implement data donation should strive to reduce participant burden. Our study shows that more burden correlates with lower participation (in line with earlier research [93]). We see that the additional steps required to request and download data from Samsung Health and especially from Google Location History come with substantially reduced data donation rates in comparison with Apple Health. Self-selection through additional burden might further contribute to differences in biases that stem from differential device brand ownership and use of these platforms. Researchers need to be aware of the multiple sources of bias when interpreting substantive results, especially in studies where data from more than 1 platform are collected. Standard adjustment procedures such as poststratification weighting based on sociodemographic variables or adding these variables as controls to regression models might need to be extended by attitudinal measures of comfort and familiarity with a specific technology to account for these differences [84,88]. Regardless of the platform, there are recommendations on how to implement data donation studies [48], which aim at improving the participants experience of the data donation process. At the same time, data donation through DDPs (ie, user-centric data donation) will have limitations imposed by data controllers who can decide on how they would like to shape their compliance with the law [94]. For the effective use of data donation for research purposes, Hase et al [95] provide a set of potential solutions to challenges that result from platforms’ decisions on how to comply with the law.

### Comparison With Prior Work

Most methodological studies around data donation have focused on the collection of usage data from social media platforms in the domain of communication research [26]. Only 3 studies so far have focused on PA-related and health-related measures: Kompier et al [62] and Toepoel et al [61] have asked individuals in the general population to donate data from their personal fitness trackers, either by uploading data from Fitbit users or by copying the information from their Garmin, Apple, and Samsung watches into the questionnaire. Silber et al [63] asked smartphone owners in the general German population to donate their health app data. To our knowledge, our study is the first to concentrate on measurement of PA using data donation from smartphones from older adults. Strikingly, we find participation rates that fall right into the middle of the range of participation rates reported in various earlier research (see section “Data Donation Participation Rates”). In general, our findings are in line with research that showed that initial stated willingness to donate data is strongly correlated with privacy concerns about the requested data [45,49,52,60,65-67]. We confirm that the actual donation conditional on consent is related to technical limitations and perceived burden [49,60,68]. In our case, requesting and donating data from Google Location History and Samsung Health included more steps than doing so from Apple Health. These additional steps might be one of the factors why we find significantly lower donation rates for Google Location History and Samsung Health.

For PA data donation, Kompier et al [62] concluded that given the low prevalence and selective group of fitness tracker owners as well as the technical challenges participants faced, data donation may not yet be a feasible way to collect PA data in the general population. Our results are more optimistic, in particular, in light of using a more user-friendly method for data donation. In the study by Kompier et al [62], respondents were asked to create a .csv data export from their wearables themselves that then had to be sent to the researchers. These steps created unrealistic technical barriers for many brands (except for Fitbit). A similar issue led to an extremely small donation rate in the study by Silber et al [63]. In this study, we requested 1 DDP and asked participants to donate through Port offering a much smoother experience for study participants. While we see selectivity in sociodemographic characteristics, the willingness to donate is high, especially among Apple smartphone owners younger than 70 years. Further optimizing the data donation process and making it more user-friendly, especially for those with low tech skills, will have the potential to provide researchers with objective PA data.

### Limitations

Older adults in our sample might be somewhat more technologically savvy than the general older population in the Netherlands and older populations in other countries. Although the LISS panel draws their sample from the Dutch population registry and non–internet users are provided with internet access and devices, having participated in monthly web surveys might have decreased the barriers of using technology in everyday life. In addition, the smartphone ownership rate in the Netherlands is quite high, although comparable with other Western European countries and the United States [36,37]. At the same time, PA data donation is feasible only for users of smartphones and the digital platforms that collect these data, and the implications of excluding certain populations should be further studied [84]. We also acknowledge that device ownership and, in particular, which brand of smartphones people own and thus from which health app they were asked to donate data were not randomly assigned in our study. We thus cannot make any causal claims about the relationship between the platform and participation rates. Furthermore, there were initially some technical problems during data collection with Google Location History DDPs that were solved timely. Nevertheless, this might have resulted in the loss of a few DDPs. Finally, not all LISS panel members invited to the survey that included the data donation request also responded. Additional analysis reported in Multimedia Appendix 3 shows that those who did not respond tend to be slightly younger and more likely to be employed than our analytical sample (Table S1 in Multimedia Appendix 3). Adjusting the results of our original model 1 predicting smartphone ownership with weights that account for these differences does not substantively change the results (Table S2 in Multimedia Appendix 3).

### Conclusions

Data donation affords researchers the opportunity to collect PA data from smartphones and digital platforms (eg, Google) that are already automatically recorded on the user’s device, thus reducing the need to design and implement lengthy and burdensome questionnaires on PA. Importantly, even when donation rates are modest, the amount of history-rich data obtained from a single donation can be substantial. Participants who donate data from Apple Health, Samsung Health, or Google Location History can often provide months or even years of retrospective data. In contrast, app-based data collection requires participants to prospectively install and maintain an application, often yielding shorter observation windows and higher dropout rates. This makes data donation not only a feasible but also an efficient and comparatively less burdensome approach for collecting large volumes of relevant behavioral data.

PA data collection via data donation could thus be of interest not only for researchers who study PA in older adults but also for government agencies and NGOs who want to determine whether citizens adhere to the World Health Organization norms. On a societal level, understanding PA patterns of older adults in their natural setting will help inform better, more targeted public policies. For example, differences in PA patterns of different groups (based on gender, race and ethnicity, socioeconomic background, and neighborhood) that will be uncovered in the donated data can inform specific policies for these groups. Our study findings contribute another step to the general understanding of whether data donation can indeed replace self-report of PA over time. In particular, we show that PA data donation from the Apple Health app on smartphones is feasible for people younger than 70 years, but bias toward a healthier sample has to be considered. A next step is to study the quality of the data provided in the DPPs to better understand the full potential of data donation to achieve true cost savings and better quality (more detailed data and higher validity as activities will not be misreported or forgotten) compared with traditional self-reports. In the meantime, PA data donation can be considered a valuable complement to self-reported PA in surveys, providing a more comprehensive picture of individuals’ PA.

## Appendix

Multimedia Appendix 1 Questionnaire.

Multimedia Appendix 2 Descriptive statistics.

Multimedia Appendix 3 Additional analysis.

Multimedia Appendix 4 Sensitivity analysis.

Multimedia Appendix 5 Analysis results.

## Abbreviations

AME: average marginal effect
DDP: Data Download Package
LISS: Longitudinal Internet studies for the Social Sciences
PA: physical activity
RQ: research question
TAM: Technology Acceptance Model
