# Supplementary material for: Data Donation as a Method to Measure Physical Activity in Older Adults: Cross-Sectional Web Survey Assessing Consent Rates, Donation Success, and Bias
Source: J Med Internet Res. 2025 Sep 26;27:e69799. doi: 10.2196/69799 (PMC12514404; doi:10.2196/69799)
Supplement: Multimedia Appendix 1 [file jmir_v27i1e69799_app1.pdf]

Multimedia Appendix 1

Questionnaire

Dutch original	English translation
Intro<br>Het doel van deze vragenlijst is om te onderzoeken hoe de dagelijkse beweging van mensen kan worden bestudeerd en of dit kan worden gedaan met behulp van technologie, zoals smartphones.<br>Eerst stellen we u enkele vragen over uw gezondheid.	 The purpose of this questionnaire is to investigate how people's daily movement can be studied and whether this can be done using technology, such as smartphones.<br>First we ask you some questions about your health.
Q1.<br>Hoe zou u over het algemeen uw gezondheid noemen?<br>1. slecht<br>2. matig<br>3. goed<br>4. zeer goed<br>5. uitstekend	 How would you describe your health, generally speaking?<br>1. bad<br>2. moderate<br>3. good<br>4. very good<br>5. excellent
Q2.<br>Hebben uw lichamelijke gezondheid of uw emotionele problemen u de<br>laatste maand belemmerd in uw alledaagse activiteiten, zoals een eindje lopen, trappen opgaan, uzelf aankleden, uzelf wassen, naar het toilet gaan?<br>1. helemaal niet<br>2. vrijwel niet<br>3. een beetje<br>4. tamelijk veel<br>5. heel erg veel	 Did your physical health or emotional problems hinder your daily activities over the past month, for instance in going for a walk, walking upstairs, dressing yourself, washing yourself, visiting the toilet?<br><br>1. not at all<br>2. hardly<br>3. a bit<br>4. quite a lot<br>5. very much
Q3.<br>Hebben uw lichamelijke gezondheid of uw emotionele problemen u de<br>laatste maand belemmerd in uw sociale activiteiten?<br>1. helemaal niet<br>2. vrijwel niet<br>3. een beetje<br>4. tamelijk veel<br>5. heel erg veel	 To what extent did your physical health or emotional problems hinder your social activities over the past month?<br><br>1. not at all<br>2. hardly<br>3. a bit<br>4. quite a lot<br>5. very much
Q4.<br>Hebben uw lichamelijke gezondheid of uw emotionele problemen u de<br>laatste maand belemmerd in uw werk, bijvoorbeeld in uw baan, in het huishouden, bij de zorg voor de kinderen, bij vrijwilligerswerk, of op school?	 To what extent did your physical health or emotional problems hinder your work over the past month, for instance in your job, the housekeeping, taking care of the children, doing volunteer work, or in school?

<table>
<tr><td>1. helemaal niet
2. vrijwel niet
3. een beetje
4. tamelijk veel
5. heel erg veel</td><td>1. not at all
2. hardly
3. a bit
4. quite a lot
5. very much</td></tr>
<tr><td>Q5.
Hieronder staan enkele handelingen, waar sommige mensen moeite mee hebben. Wilt u voor elke handeling aangeven of u die zonder moeite, met enige moeite, met grote moeite, of alleen met hulp van anderen kunt doen? Het gaat hier niet om problemen waarvan u verwacht dat ze korter dan drie maanden zullen duren.

Q5_1 100 meter lopen
Q5_2 ongeveer twee uur lang zitten
Q5_3 opstaan uit een stoel als u langere tijd hebt gezeten
Q5_4 meerdere trappen oplopen zonder te rusten
Q5_5 een trap oplopen zonder te rusten
Q5_6 hurken, knielen en kruipen

Q5_7 boven schouderhoogte reiken of uw armen boven schouderhoogte uitstrekken
Q5_8 grote voorwerpen verplaatsen zoals een eetkamerstoel
Q5_9 een gewicht van 5 kilo optillen of dragen, zoals een zware tas met boodschappen
Q5_10 een klein muntje oppakken van een tafel
1. zonder moeite
2. met enige moeite
3. met grote moeite
4. alleen met hulp van anderen
5. niet</td><td>
Below you will find a number of actions that some people have difficulties with. Can you indicate, for each activity, whether you can perform it without any trouble, with some trouble, with a lot of trouble, only with the help of others or not at all? This question does not apply to problems which you expect will not last longer than three months.
Q5_1 walking 100 meters
Q5_2 sitting for around two hours
Q5_3 getting up from a chair in which you sat for some time
Q5_4 walking several stairs without resting in between
Q5_5 walking up a staircase without resting
Q5_6 crouching, kneeling, crawling on all fours
Q5_7 reaching above shoulder height or stretching your arms above shoulder height
Q5_8 moving large objects such as a dining room chair
Q5_9 lifting or carrying a weight of 5 kilos, such as a heavy bag of groceries

Q5_10 picking up a small coin lying on the table
1. without any trouble
2. with some trouble
3. with a lot of trouble
4. only with an aid or the help of others
5. not</td></tr>
<tr><td>Q6.
Hebt u één of meer langdurige ziekten of aandoeningen?
Antwoordtype: Keuzerondjes
1. Ja
2. Nee
3. Dat zeg ik liever niet</td><td>
Do you have one or more long-term illnesses or conditions?
Answer type: Radio buttons
1. Yes
2. No
3. I'd rather not say that</td></tr>
<tr><td>Intro</td><td></td></tr>
</table>

Wij zijn geïnteresseerd welke lichamelijke activiteiten mensen verrichten in hun dagelijkse leven. De volgende vragen gaan over uw lichamelijke activiteit gedurende de afgelopen 7 dagen. Denkt u aan activiteiten die u doet op het werk, in en rond het huis, om van de ene naar de andere plaats te komen, en activiteiten in uw vrije tijd voor recreatie, training of sport. Denkt u aan alle zware lichamelijke activiteiten die u deed in de afgelopen 7 dagen. Zware lichamelijke activiteiten zijn activiteiten die veel lichamelijke inspanning kosten en voor een veel snellere ademhaling zorgen. Denk alleen aan de activiteiten die u ten minste 10 minuten per keer hebt verricht.	We are interested in what physical activity people perform in their daily lives. The following questions are about your physical activity over the past 7 days. Think of activities that you perform at work, in and around the house, to get from one place to another, and activities in leisure time relating to recreation, training or sports.Think of all the strenuous activities you performed these last 7 days. Strenuous physical activities are activities that require a great deal of physical exertion and cause you to breathe a lot faster than normally. Only consider those activities that you performed for at least 10 minutes per occasion.
Q7.<br>Als u denkt aan de afgelopen 7 dagen, op hoeveel van deze dagen hebt u zware lichamelijke activiteiten verricht zoals zware lasten tillen, spitten, aerobics of wielrennen? Denk alleen aan de activiteiten die u ten minste 10 minuten per keer hebt verricht.<br>________	If you look back on the last 7 days, on how many of those days did you perform a strenuous physical activity such as lifting heavy loads, digging, aerobics or cycling? If you did not perform any strenuous physical activity, enter zero (0).<br>_______
ASK IF Q7>0<br>*Q8.*<br>Op de dagen dat u zwaar lichamelijk actief was, hoeveel tijd hebt u daar dan gewoonlijk aan besteed?<br>___________aantal uren per dag<br>___________aantal minuten per dag	On the days that you performed a strenuous physical activity, how much time did you usually spend on this activity? You can enter your answer as an average number of hours and minutes per day.<br>___________number of hours per day<br>___________number of minutes per day
Q9.<br>Denkt u aan activiteiten die matige lichamelijke inspanning kosten en die u in de afgelopen 7 dagen hebt verricht. Matig intensieve lichamelijke activiteit laat u iets sneller ademen dan normaal. Denkt u weer alleen aan activiteiten die u ten minste 10 minuten per keer hebt verricht. Als u denkt aan de afgelopen 7 dagen, op hoeveel van deze dagen hebt u matig intensieve lichamelijke activiteit verricht, zoals het dragen van lichte lasten, fietsen in een normaal tempo of ramen zemen? Laat wandelen hier buiten beschouwing, daar krijgt u straks een vraag over<br>_______	Think of activities that you performed over the last 7 days that require moderate physical exertion. Moderately intensive physical activities cause you to breathe somewhat faster than normally. Again, think only of activities that you performed for at least 10 minutes per occasion. If you think of the past 7 days, on how many of those days did you perform a moderately intensive physical activity such as carrying light loads, cycling at a normal pace or cleaning windows? If you did not perform moderately intensive physical activities, enter zero (0).<br>_______

ASK IF Q9>0<br>*Q10.*<br>Op de dagen dat u matig intensief lichamelijk actief was, hoeveel tijd hebt u daar dan gewoonlijk aan besteed?<br>___________aantal uren per dag<br>___________aantal minuten per dag	On the days that you performed a moderately intensive physical activity, how much time did you usually spend on this activity? You can enter your answer as an average number of hours and minutes per day.<br>___________number of hours per day<br>___________number of minutes per day
Q11.<br>Als u denkt aan de afgelopen 7 dagen, op hoeveel dagen hebt u ten minste 10 minuten per keer gewandeld? Denk hierbij aan wandelen op het werk en thuis, wandelen om van de ene naar de andere plaats te komen, en al het andere wandelen dat u deed tijdens recreatie, sport of vrijetijdsbesteding. Als u niet of minder dan 10 minuten hebt gewandeld kunt u een 0 invullen.<br>______	If you look back on the last 7 days, on how many of those days did you spend at least 10 minutes walking? Think of walking on the job and at home, walking to get from one place to another, and all the walking you did as part of recreation, sports or leisure time activities. If you did not walk or walked for less than 10 minutes, enter zero (0).<br>______
ASK IF Q11>0<br>*Q12.*<br>Op de dagen dat u ten minste 10 minuten per keer wandelde, hoeveel tijd hebt u daar dan gewoonlijk aan besteed? U kunt uw antwoord invullen in een gemiddeld aantal uren en minuten per dag (60 minuten= 1 uur).<br>___________aantal uren per dag<br>___________aantal minuten per dag	On the days that you spent at least 10 minutes walking per occasion, how much time did you usually spend on this? You can enter your answer as an average number of hours and minutes per day (60 minutes = 1 hour).<br>___________number of hours per day<br>___________number of minutes per day
Q13.<br>Als u terugkijkt op de afgelopen 7 dagen, op hoeveel van die dagen heeft u dan minimaal 10 minuten gefietst? Denk aan fietsen naar je werk, fietsen om van de ene plaats naar de andere te komen, en al het fietsen dat je hebt gedaan als onderdeel van recreatie, sport of vrijetijdsbesteding. Heeft u minder dan 10 minuten gefietst of niet gefietst, vul dan nul (0) in.<br>______	If you look back on the last 7 days, on how many of those days did you spend at least 10 minutes biking? Think of biking to work, biking to get from one place to another, and all the biking you did as part of recreation, sports or leisure time activities. If you did not bike or biked for less than 10 minutes, enter zero (0).<br>______
ASK IF Q13>0<br>*Q14.*<br>Op de dagen dat u per dag minimaal 10 minuten fietste, hoeveel tijd besteedde u daar doorgaans per dag aan? U kunt uw antwoord invoeren als een gemiddeld aantal uren en minuten	On the days that you spent at least 10 minutes biking per occasion, how much time did you usually spend on this? You can enter your answer as an average number of

___________aantal uren per dag<br>___________aantal minuten per dag	hours and minutes per day (60 minutes = 1 hour).<br>___________number of hours per day<br>___________number of minutes per day
Q15.<br>Als u terugkijkt op de afgelopen 7 dagen, op hoeveel van die dagen heeft u dan minstens 10 minuten hardgelopen? Denk aan hardlopen als onderdeel van recreatie, sport of vrijetijdsbesteding. Als u niet of minder dan 10 minuten heeft hardgelopen, vult u nul (0) in.<br>______	If you look back on the last 7 days, on how many of those days did you spend at least 10 minutes running? Think of running as part of recreation, sports or leisure time activities. If you did not run or ran for less than 10 minutes, enter zero (0).<br>______
ASK IF Q15>0<br>*Q16.*<br>Op de dagen dat u minimaal 10 minuten aan hardlopen besteedde, hoeveel tijd besteedde u daar doorgaans per dag aan? U kunt uw antwoord invoeren als een gemiddeld aantal uren en minuten per dag (60 minuten = 1 uur).<br>___________aantal uren per dag<br>___________aantal minuten per dag	On the days that you spent at least 10 minutes running per occasion, how much time did you usually spend on this? You can enter your answer as an average number of hours and minutes per day (60 minutes = 1 hour).<br>___________number of hours per day<br>___________number of minutes per day
Q17.<br>Hoeveel tijd bracht u gewoonlijk zittend door gedurende een doordeweekse dag in de afgelopen 7 dagen? Bij deze tijd mag zitten achter een bureau of computer, tijd die zittend wordt doorgebracht met vrienden, zittend lezen, studeren, internetten of tv kijken worden gerekend. U kunt uw antwoord invullen in een gemiddeld aantal uren en minuten per dag (60 minuten= 1 uur).<br>___________aantal uren per dag<br>___________aantal minuten per dag	How much time did you usually spend seated during a normal week day, over the past 7 days? This time can include time spent seated at a desk or behind a computer, seated while socializing with friends, seated while reading, studying, using the internet or watching TV. You can enter your answer as an average number of hours and minutes per day (60 minutes = 1 hour).<br>___________number of hours per day<br>___________number of minutes per day
Q18.<br>Bent u gisteren buiten geweest?<br>We bedoelen hier niet uw tuin of balkon, maar echt de deur uit<br>1. Ja<br>2. Nee	Were you outside yesterday?<br>We don't mean your garden or balcony here, but really out the door<br>1. Yes<br>2. No
IF Q18=1, ASK Q19, ELSE Q21<br>*Q19.*<br>Hoeveel kilometer hebt u **gisteren** afgelegd? Als u het niet precies weet, geef dan een schatting.	How many kilometers did you travel yesterday? If you don't know exactly, please give an estimate.

We bedoelen hier de afstand van alle manieren van buitenshuis reizen bij elkaar opgeteld (zoals lopen, fietsen, met de auto en openbaar vervoer).<br>_______	Here we mean the distance of all ways of traveling outdoors added together (such as walking, cycling, by car and public transport).<br>_______
Q20.<br>Hoeveel tijd was u gisteren buitenshuis onderweg? Als u het niet precies weet, geef dan een schatting.<br>We bedoelen hier de tijd van alle manieren van buitenshuis reizen bij elkaar opgeteld (zoals lopen, fietsen, met de auto en openbaar vervoer).<br>_______	How much time did you spend outdoors yesterday? If you don't know exactly, please give an estimate.<br>Here we mean the time of all ways of traveling outdoors added together (such as walking, cycling, by car and public transport).<br>_______
Q21.<br>Hoe lang bent u?<br>_______ cm	How tall are you?<br>_______ cm
Q22.<br>Hoeveel weegt u zonder kleren en schoenen?<br>_______ kilo	How much do you weigh without clothes and shoes?<br>_______ kilo
Q23.<br>Volgens onze eerder verzamelde gegevens hebt u een smartphone. Hebt u nog steeds een smartphone?<br>1. Ja<br>2. Nee	According to our previously collected data, you have a smartphone. Do you still have a smartphone?<br>1. Yes<br>2. No
ASK IF Q23 = 1<br>*Q24.*<br>We hebben een paar vragen over uw dagelijks gebruik van technologie.<br>Wat voor smartphone gebruikt u?<br>Als u meerdere smartphones bezit, beantwoord dan de vraag over de smartphone die u het meest gebruikt.<br>1. iPhone<br>2. Android Phone (bijv. Samsung, Huawei, LG, Motorola, Sony)<br>3. Windows Phone<br>4. Blackberry<br>5. Anders<br>6. Weet ik niet/Ik twijfel	We have a couple of questions about your everyday use of technology.<br>What kind of smartphone do you use?<br>If you own multiple smartphones, answer the question about the smartphone you use most.<br>1. iPhone<br>2. Android Phone (for example Samsung, Huawei, LG, Motorola, Sony)<br>3. Windows Phone<br>4. Blackberry<br>5. Different<br>6. I don't know/I have doubts
ASK IF Q24 = 1<br>Q25<br>Gebruikt u uw smartphone voor de volgende activiteiten?<br>Q25a Het sturen van berichten (bijvoorbeeld met sms, Whatsapp of Telegram)	Do you use your smartphone for the following activities?<br>Q25a Sending messages (for example with SMS, WhatsApp or Telegram)

Q25b Websites bezoeken<br>Q25c Het bekijken of schrijven van e-mails<br>Q25d Het nemen van foto's<br>Q25e Het maken van videos<br>Q25f Het bekijken van social media (bijvoorbeeld berichtjes, foto's, video's op Facebook, Twitter, Instagram)<br>Q25g Het posten op social media (bijvoorbeeld berichtjes, foto's, video's op Facebook, Twitter, Instagram enz.)<br>Q25h Het doen van aankopen (bijvoorbeeld het kopen van boeken of kleding, treinkaartjes, bestellen van eten)<br>Q25i Internetbankieren (bijvoorbeeld het bekijken van uw saldo of geld overmaken)<br>Q25j Het installeren van nieuwe apps (bijvoorbeeld via de App Store van Apple, Google Play Store)<br>Q25k Het gebruiken van GPS/locatie-apps (bijvoorbeeld Google Maps, Foursquare, Yelp)<br>Q25l Het verbinden met andere elektronische apparaten via Bluetooth (bijvoorbeeld smartwatches, fitness armbanden, stappentellers)<br>Q25m Bellen (ook via bv Skype of Facetime)<br>Q25n Het spelen van spelletjes<br>Q25o Het streamen van muziek of video<br>Q25p Anders<br>1. Ja<br>2. Nee	Q25b Visit websites<br>Q25c Viewing or writing emails<br>Q25d Taking photos<br>Q25e Making videos<br>Q25f Viewing social media (e.g. messages, photos, videos on Facebook, Twitter, Instagram)<br>Q25g Posting on social media (e.g. messages, photos, videos on Facebook, Twitter, Instagram, etc.)<br>Q25h Making purchases (e.g. buying books or clothes, train tickets, ordering food)<br>Q25i Internet banking (for example viewing your balance or transferring money)<br>Q25j Installing new apps (for example via the Apple App Store, Google Play Store)<br>Q25k Using GPS/location apps (e.g. Google Maps, Foursquare, Yelp)<br>Q25l Connecting to other electronic devices via Bluetooth (e.g. smartwatches, fitness bracelets, pedometers)<br>Q25m Calling (also via Skype or Facetime)<br>Q25n Playing games<br>Q25o Streaming music or video<br>Q25p Other<br>1. Yes<br>2. No
Q26.<br>Hoe bezorgd bent u over het algemeen over uw privacy?<br>1. Helemaal niet bezorgd<br>2. Niet erg bezorgd<br>3. Een beetje bezorgd<br>4. Zeer bezorgd	How concerned are you about your privacy in general?<br>1. Not concerned at all<br>2. Not very concerned<br>3. A little worried<br>4. Very concerned
Q27.<br>*De items zijn in willekeurige volgorde aangeboden (zie Q27_permutatie__1 – Q27_permutatie__4).*<br>Geef aan hoe privé de volgende informatie voor u voelt:<br>Q27a Mijn huidige locatie<br>Q27b Mijn dagelijks reisgedrag<br>Q27c Mijn lichamelijke activiteit (bijvoorbeeld hoeveel ik loop)<br>Q27d Mijn gezondheidsgegevens<br>1. 1 Helemaal niet privé	*The items are presented in random order (see Q27_permutation__1 – Q27_permutation__4).*<br>Please indicate how private the following information feels to you:<br>Q27a My current location<br>Q27b My daily travel behavior<br>Q27c My physical activity (e.g. how much I walk)<br>Q27d My health data<br>1. 1 Not private at all

2. 2<br>3. 3<br>4. 4<br>5. 5 Zeer privé	2. 2<br>3. 3<br>4. 4<br>5. 5 Very private
Q28.<br>Tegenwoordig worden er veel persoonlijke data verzameld. In hoeverre vertrouwt u de volgende organisaties dat zij uw persoonlijke informatie niet delen met anderen?<br>Vraagtype: Tabel Antwoordtype: Keuzerondjes Subvragen:<br>Q28a Publieke opiniepeilers en marktonderzoekbureaus, zoals Ipsos of Kantar<br>Q28b Wetenschappelijke onderzoekers<br>Q28c Overheidsinstellingen, zoals gemeente, nationale overheid<br>Q28d Officiële statistiekinstituten, zoals het CBS<br>Q28e Bedrijven die apps voor smartphones maken<br>Q28f Onlinewinkels, zoals Amazon of bol.com<br>Q28g Social-mediabedrijven, zoals Facebook, Twitter en LinkedIn<br>Q28h Technologiebedrijven, zoals Google<br><br>1. Ik vertrouw ze helemaal niet<br>2. Ik vertrouw ze weinig<br>3. Ik vertrouw ze een beetje<br>4. Ik vertrouw ze volledig	 Nowadays a lot of personal data is collected. To what extent do you trust the following organizations not to share your personal information with others?<br><br>Question type: Table Answer type: Radio buttons Sub-questions:<br>Q28a Public opinion pollsters and market research agencies, such as Ipsos or Kantar<br><br>Q28b Scientific researchers<br>Q28c Government institutions, such as municipality, national government<br>Q28d Official statistical institutes, such as Statistics Netherlands<br>Q28e Companies that make apps for smartphones<br>Q28f Online stores, such as Amazon or bol.com<br>Q28g Social media companies, such as Facebook, Twitter and LinkedIn<br>Q28h Technology companies, such as Google<br>1. I don't trust them at all<br>2. I don't trust them much<br>3. I trust them a little<br>4. I trust them completely
Data donation request<br>U hebt in de vragen tot nu toe aangegeven welke vormen van lichamelijke activiteit u heeft gehad. Samen met de Universiteit Utrecht onderzoeken we of lichamelijke activiteit op een andere manier kunnen worden onderzocht, bijvoorbeeld door het gebruik van technologie zoals smartphones. We vragen u daarom mee te doen aan een studie naar het gebruik van technologie om de dagelijkse lichamelijke activiteit/beweging van mensen te bestuderen. We leggen op de volgende schermen eerst uit wat de studie inhoudt en hoe het werkt, zodat u daarna kunt beslissen of u wel of niet wilt meedoen	 You have indicated which forms of physical activity you have done in the questions so far. Together with Utrecht University, we are investigating whether physical activity can be investigated in a different way, for example by using technology such as smartphones. We therefore ask you to participate in a study into the use of technology to study people's daily physical activity/exercise. On the following screens we first explain what the study entails and how it works, so that you can then decide whether or not you want to participate
IF ANDROID:	

Op sommige smartphones verzamelt Google informatie over bewegingen. Deze bewegingen worden onthouden door de Google Locatie Geschiedenis en Google Fit. Het doel van de studie is om de Google Locatie Geschiedenis en Google Fit op te vragen bij Google. Zo kan onderzocht worden of op deze manier lichamelijke bewegingen bestudeerd kunnen worden. Als u de Google Locatie Geschiedenis en/of Google Fit opvraagt stuurt Google u een pakketje (bestand) toe via e-mail, dat u opslaat op uw computer. Uit dit pakketje kunnen de bewegingen worden gehaald die van belang zijn voor de studie. Bijvoorbeeld hoeveel tijd u tussen 2018 en 2023 heeft gefietst, gelopen of gejogd of hoeveel stappen u gelopen hebt. Dat hoeft u niet zelf te doen, dit gebeurt automatisch met speciaal voor deze studie ontwikkelde software. Privacy is hierbij gewaarborgd, omdat er alleen het type bewegingen en geen persoonlijke gegevens uit het pakketje worden gehaald. [EXP1: Een voorbeeld van hoe deze informatie eruit ziet ziet u hieronder]. De informatie over bewegingen kunt u vervolgens in deze vragenlijst delen met Centerdata. Het is daarbij goed om te weten dat locaties die u hebt bezocht niet uit het pakketje worden gehaald en dus ook niet met Centerdata worden gedeeld. Er wordt alleen informatie gedeeld hoe u zich heeft bewegt en hoeveel tijd u hieraan heeft besteed per dag. U ziet vooraf welke informatie uit het pakketje is gehaald. U kunt dan alsnog beslissen of u de informatie wel of niet wilt delen met Centerdata.	Google collects information about movements on some smartphones. These movements are remembered by Google Location History and Google Fit. The purpose of the study is to request Google Location History and Google Fit from Google. This way it can be investigated whether movements can be studied in this way. If you request Google Location History and/or Google Fit, Google will send you a package (file) via email, which you save on your computer. The movements that are important for the study can be extracted from this package. For example, how much time you cycled, walked or jogged between 2018 and 2023 or how many steps you walked. You do not have to do this yourself, this is done automatically with software specially developed for this study. Privacy is guaranteed, because only the type of movements and no personal data are extracted from the package. [EXP1: An example of what this information looks like is shown below]. You can then share the information about movements in this questionnaire with Centerdata. It is good to know that locations you have visited are not removed from the package and are therefore not shared with Centerdata. Information is only shared about how you exercised and how much time you spent on it per day. You can see in advance what information has been extracted from the package. You can then still decide whether or not you want to share the information with Centerdata.
IF IPHONE:	

Op sommige smartphones verzamelt Apple informatie over bewegingen. Deze bewegingen worden onthouden door de iHealth App op een iPhone. Het doel van de studie is om de iHealth gegevens op te vragen bij Apple. Zo kan onderzocht worden of op deze manier bewegingen bestudeerd kunnen worden. Als u de iHealth informatie opvraagt stuurt Apple u een pakketje (bestand) toe via e-mail, dat u opslaat op uw computer. Uit dit pakketje kunnen de bewegingen worden gehaald die van belang zijn voor de studie. Bijvoorbeeld hoeveel stappen u tussen 2018 en 2023 hebt gelopen of gejogd. Dat hoeft u niet zelf te doen, dit gebeurt automatisch met speciaal voor deze studie ontwikkelde software. Privacy is hierbij gewaarborgd, omdat er alleen de aantal stappen en kilometer en geen persoonlijke gegevens uit het pakketje worden gehaald. [EXP1: Een voorbeeld van hoe deze informatie eruitziet ziet u hieronder]. De informatie over lichamelijke bewegingen kunt u vervolgens in deze vragenlijst delen met Centerdata. U ziet vooraf welke informatie uit het pakketje is gehaald. U kunt dan alsnog beslissen of u de informatie wel of niet wilt delen met Centerdata.	On some smartphones, Apple collects information about movements. These movements are remembered by the iHealth App on an iPhone. The purpose of the study is to request iHealth data from Apple. This way it can be investigated whether movements can be studied in this way. If you request iHealth information, Apple will send you a package (file) via email, which you save on your computer. The movements that are important for the study can be extracted from this package. For example, how many steps you walked or jogged between 2018 and 2023. You do not have to do this yourself, this is done automatically with software specially developed for this study. Privacy is guaranteed, because only the number of steps and kilometers and no personal data are extracted from the package. [EXP1: An example of what this information looks like is shown below]. You can then share the information about movements in this questionnaire with Centerdata. You can see in advance what information has been extracted from the package. You can then still decide whether or not you want to share the information with Centerdata.
Q29.<br>Bent u bereid om deze gegevens met Centerdata te delen?<br>1. Ja<br>2. Nee	 Are you willing to share this data with Centerdata?<br>1. Yes<br>2. No
IF Q29 = 2<br>*Q30.*<br>Wat zijn de redenen dat u deze gegevens niet wilt delen?<br>________________________________	 <br>What are the reasons that you do not want to share this data?<br>________________________________
Q31.<br><br>We willen graag weten of de uitleg van de studie duidelijk was. De volgende stellingen gaan over de uitleg van de studie. Geeft u voor elke aan of u deze juist of onjuist vindt. Dit betreft geen toetsing en u hoeft de vorige vraag dan ook niet opnieuw te bekijken.	 <br>We would like to know whether the explanation of the study was clear. The following statements are about the explanation of the study. Please indicate for each whether you think it is correct or incorrect. This is not an assessment and you do not need to revisit the previous question.

Houdt u het proces om de informatie over uw gegevens op te vragen en met Centerdata te delen in gedachten:<br>Q31a. U wordt gevraagd om informatie bij [Google] [Apple] op te vragen.<br>Q31b. De software die voor dit onderzoek wordt gebruikt filtert de informatie over hoe veel [tijd u fietst, loopt, of jogt] [stappen u loopt].<br>Q31c. Informatie over alle plekken die u hebt bezocht zal met Centerdata worden gedeeld.<br>Q31d. U kunt worden geïdentificeerd op basis van de informatie die u deelt.<br>Q31e. U kunt de informatie eerst inzien voordat u deze met Centerdata deelt.<br>1. juist<br>2. onjuist<br>3. weet ik niet	Please keep in mind the process for requesting and sharing information about your data with Centerdata:<br>Q31a. You will be asked to request information from [Google][Apple].<br>Q31b. The software used for this study filters the information about how much [time you cycle, walk, or jog] [steps you walk].<br>Q31c. Information about all the places you have visited will be shared with Centerdata.<br>Q31d. You can be identified based on the information you share.<br>Q31e. You can first view the information before sharing it with Centerdata.<br>1. correct<br>2. incorrect<br>3. I don't know
