# Supplementary material for: Data Donation as a Method to Measure Physical Activity in Older Adults: Cross-Sectional Web Survey Assessing Consent Rates, Donation Success, and Bias
Source: J Med Internet Res. 2025 Sep 26;27:e69799. doi: 10.2196/69799 (PMC12514404; doi:10.2196/69799)
Supplement: Multimedia Appendix 2 [file jmir_v27i1e69799_app2.pdf]

Multimedia Appendix 2

Table S1. Descriptive statistics of categorical variables.

Variables		n (%)
**Gender**		
	Female	1045 (50.1%)
	Male	1041 (49.9%)
	NA	0
**Age**		
	50-54 years	284 (13.6%)
	55-59 years	296 (14.2%)
	60-64 years	368 (17.6%)
	65-69 years	339 (16.3%)
	70-74 years	341 (16.4%)
	75-79 years	266 (12.8%)
	80 years and older	192 (9.2%)
	NA	0
**HH size**		
	Single-person HH	531 (25.5%)
	Two-person HH	1,146 (54.9%)
	Three- and more person HH	409 (19.6%)
	NA	0
**Urbanicity**		
	Not urban	380 (18.2%)
	Little urban	599 (28.7%)
	Moderately urban	425 (20.4%)
	Strongly urban	381 (18.3%)
	Very strongly urban	299 (14.3%)
	NA	2
**Employment status**		
	Employed for pay	794 (38.1%)
	Unpaid work, incl. housework	250 (12.0%)
	Unemployed, retired, or disabled	1,042 (50.0%)
**Monthly personal net income**		
	Up to EUR 1,000	255 (10.8%)
	EUR 1,001 – EUR 1,500	310 (14.9%)
	EUR 1,501 – EUR 2,000	340 (16.3%)
	EUR 2,001 – EUR 2,500	325 (15.6%)
	EUR 2,501 – EUR 3,000	267 (12.8%)
	More than EUR 3,000	426 (20.4%)
	No income/NA	193 (9.3%)
**Educational attainment**		
	Low	569 (27.3%)
	Medium	710 (34.1%)
	High	803 (38.6%)
	NA	4
**General privacy concerns**		
	Not at all concerned	174 (8.3%)
	Not very concerned	712 (34.1%)

	A little concerned	1008 (48.3%)
	Very concerned	191 (9.2%)
	NA	1
**Perceived privacy of current location data**		
	1 Not at all private	228 (10.9%)
	2	229 (11.0%)
	3	565 (27.1%)
	4	593 (28.4%)
	5 Very private	470 (22.5%)
	NA	1
**Perceived privacy of daily travel behavior data**		
	1 Not at all private	283 (13.6%)
	2	301 (14.4%)
	3	669 (32.1%)
	4	541 (25.9%)
	5 Very private	291 (14.0%)
	NA	1
**Perceived privacy of physical activity data**		
	1 Not at all private	283 (13.6%)
	2	300 (14.4%)
	3	644 (30.9%)
	4	529 (25.4%)
	5 Very private	329 (15.8%)
	NA	1
**Perceived privacy of personal health data**		
	1 Not at all private	125 (6.0%)
	2	158 (7.6%)
	3	518 (24.8%)
	4	603 (28.9%)
	5 Very private	681 (32.7%)
	NA	1
**Perceived privacy of information (Index terciles)**		
	Low	695 (33.3%)
	Medium	695 (33.3%)
	High	695 (33.3%)
	NA	1
**Trust in pollsters and market research agencies**		
	I don't trust them at all	420 (20.2%)
	I don't trust them much	762 (36.6%)
	I trust them a little	787 (37.8%)
	I trust them completely	115 (5.5%)
	NA	1
**Trust in scientific researchers**		
	I don't trust them at all	86 (4.1%)
	I don't trust them much	311 (14.9%)
	I trust them a little	1,123 (53.9%)
	I trust them completely	564 (27.1%)
	NA	2
**Trust in government institutions**		

	I don't trust them at all	143 (6.9%)
	I don't trust them much	459 (22.0%)
	I trust them a little	1,119 (53.7%)
	I trust them completely	363 (17.4%)
	NA	2
**Trust in official statistical institutes**		
	I don't trust them at all	108 (5.2%)
	I don't trust them much	356 (17.1%)
	I trust them a little	1,090 (52.3%)
	I trust them completely	530 (25.4%)
	NA	2
**Trust in companies that make apps**		
	I don't trust them at all	500 (24.0%)
	I don't trust them much	978 (46.9%)
	I trust them a little	572 (27.4%)
	I trust them completely	34 (1.6%)
	NA	2
**Trust in online stores**		
	I don't trust them at all	484 (23.2%)
	I don't trust them much	759 (36.4%)
	I trust them a little	737 (35.4%)
	I trust them completely	104 (5.0%)
	NA	2
**Trust in social media companies**		
	I don't trust them at all	875 (42.0%)
	I don't trust them much	807 (38.7%)
	I trust them a little	387 (18.6%)
	I trust them completely	15 (0.7%)
	NA	2
**Trust in technology companies**		
	I don't trust them at all	615 (29.5%)
	I don't trust them much	845 (40.5%)
	I trust them a little	582 (27.9%)
	I trust them completely	42 (2.0%)
	NA	2
**Trust in government & research institutions (Index terciles)**		
	Low	695 (33.3%)
	Medium	695 (33.3%)
	High	694 (33.3%)
	NA	2
**Trust in technology companies (Index terciles)**		
	Low	695 (33.3%)
	Medium	695 (33.3%)
	High	694 (33.3%)
	NA	2
**Self-rated health (original)**		
	Bad	38 (1.8%)
	Moderate	437 (22.7%)
	Good	1,202 (57.6%)

	Very good	309 (14.8%)
	Excellent	64 (3.1%)
	NA	0
**Self-rated health (recoded)**		
	Moderate/Bad	511 (24.5%)
	Good	1,202 (57.6%)
	Excellent/Very good	373 (17.9%)
	NA	0
**Chronic illness**		
	No	1,160 (56.9%)
	Yes	879 (43.1%)
	NA	47
**BMI (original)**		
	Underweight	13 (0.6%)
	Healthy weight	870 (41.9%)
	Overweight	826 (39.8%)
	Obesity	365 (17.6%)
	NA	12
**BMI (recoded)**		
	Underweight/Healthy weight	883 (42.6%)
	Overweight	826 (39.8%)
	Obesity	365 (17.6%)
	NA	12
**Limited by health in daily activities**		
	Not at all	1,017 (48.8%)
	Hardly	418 (20.0%)
	A bit	408 (19.6%)
	Quite a lot	204 (9.8%)
	Very much	39 (1.9%)
	NA	0
**Limited by health in social activities**		
	Not at all	1,090 (52.3%)
	Hardly	461 (22.1%)
	A bit	355 (17.0%)
	Quite a lot	148 (7.1%)
	Very much	32 (1.5%)
	NA	0
**Limited by health in work**		
	Not at all	1,108 (53.1%)
	Hardly	398 (19.1%)
	A bit	372 (17.8%)
	Quite a lot	158 (7.6%)
	Very much	50 (2.4%)
	NA	0
**Limited by health in activities (Index terciles)**		
	Low	698 (33.5%)
	Medium	695 (33.3%)
	High	693 (33.2%)
	NA	0

**Walking 100 meters**		
	Without any difficulties	1,713 (82.1%)
	With some difficulties	257 (12.3%)
	With a lot of difficulties	60 (2.9%)
	Only with an aid or the help of others	32 (1.5%)
	Not	24 (1.2%)
	NA	0
**Sitting for around two hours**		
	Without any difficulties	1,563 (74.9%)
	With some difficulties	433 (20.8%)
	With a lot of difficulties	72 (3.5%)
	Only with an aid or the help of others	2 (0.1%)
	Not	16 (0.8%)
	NA	0
**Getting up from chair**		
	Without any difficulties	1,269 (60.8%)
	With some difficulties	711 (34.1%)
	With a lot of difficulties	89 (4.3%)
	Only with an aid or the help of others	9 (0.4%)
	Not	8 (0.4%)
	NA	0
**Walking several stairs without resting**		
	Without any difficulties	1,087 (52.1%)
	With some difficulties	688 (33.0%)
	With a lot of difficulties	248 (11.9%)
	Only with an aid or the help of others	11 (0.5%)
	Not	52 (2.5%)
	NA	0
**Walking up staircase without resting**		
	Without any difficulties	1,555 (74.5%)
	With some difficulties	358 (17.2%)
	With a lot of difficulties	122 (5.8%)
	Only with an aid or the help of others	12 (0.6%)
	Not	39 (1.9%)
	NA	0
**Crouching, kneeling, crawling**		
	Without any difficulties	936 (44.9%)
	With some difficulties	746 (35.8%)
	With a lot of difficulties	288 (13.8%)
	Only with an aid or the help of others	33 (1.6%)
	Not	83 (4.0%)
	NA	0
**Reaching above shoulder height**		
	Without any difficulties	1,496 (71.7%)
	With some difficulties	448 (21.5%)
	With a lot of difficulties	104 (5.0%)
	Only with an aid or the help of others	12 (0.6%)
	Not	26 (1.2%)

	NA	0
**Moving large objects**		
	Without any difficulties	1,464 (70.2%)
	With some difficulties	460 (22.1%)
	With a lot of difficulties	111 (5.3%)
	Only with an aid or the help of others	22 (1.1%)
	Not	29 (1.4%)
	NA	0
**Lifting or carrying 5 kilos**		
	Without any difficulties	1,421 (68.1%)
	With some difficulties	461 (22.1%)
	With a lot of difficulties	142 (6.8%)
	Only with an aid or the help of others	22 (1.1%)
	Not	40 (1.9%)
	NA	0
**Picking up small coin from table**		
	Without any difficulties	1,886 (90.4%)
	With some difficulties	154 (7.4%)
	With a lot of difficulties	38 (1.8%)
	Only with an aid or the help of others	1 (0.1%)
	Not	7 (0.3%)
	NA	0
**Difficulties with tasks (Index terciles)**		
	Low	698 (33.5%)
	Medium	695 (33.3%)
	High	693 (33.2%)
	NA	0
**No. days with moderate physical activity (Terciles)**		
	Low	696 (33.4%)
	Medium	696 (33.4%)
	High	694 (33.3%)
	NA	0
**No. days with strenuous physical activity (Terciles)**		
	Low	694 (33.3%)
	Medium	695 (33.3%)
	High	697 (33.4%)
	NA	0
**No. days walking (Terciles)**		
	Low	695 (33.3%)
	Medium	695 (33.3%)
	High	696 (33.4%)
	NA	0
**No. days biking (Terciles)**		
	Low	694 (33.3%)
	Medium	696 (33.4%)
	High	696 (33.4%)
	NA	0
**No. days running (Terciles)**		
	Low	695 (33.3%)

	Medium	695 (33.3%)
	High	696 (33.4%)
	NA	0
**Time sedentary in h (Terciles)**		
	Low	695 (33.3%)
	Medium	695 (33.3%)
	High	696 (33.4%)
	NA	0
**Spending time outdoors yesterday**		
	No	324 (15.5%)
	Yes	1,762 (84.5%)
	NA	0

Table S2. Descriptive statistics of continuous variables.

Variable	n	Mean	Median	SD	NA
Perceived privacy of information (Index)	2,086	3.4	3.5	1.00	1
Trust in government & research institutions (Index)	2,086	2.8	2.8	0.65	2
Trust in technology companies (Index)	2,086	2.0	2.0	0.68	2
BMI	2,086	26.4	25.7	4.61	12
Limited in activities (Index)	2,086	1.9	1.7	1.00	0
Difficulties with tasks (Index)	2,086	1.4	1.2	0.56	0
No. days with moderate physical activity	2,086	3.5	3.0	2.52	0
No. days with strenuous physical activity	2,086	1.2	0	1.83	0
No. days walking	2,086	4.4	5.0	2.57	0
No. days biking	2,086	1.9	1.0	2.23	0
No. days running	2,086	0.1	0	0.6	0
Time sedentary in h	2,086	6.3	6.0	3.27	0

Table S3. Exploratory factor analysis: perceived privacy of information—Kaiser-Meyer-Olkin (KMO) test.

Variable		MSA
Overall		.78
	Perceived privacy of current location data	.78
	Perceived privacy of travel behavior data	.73
	Perceived privacy of physical activity data	.79
	Perceived privacy of personal health data	.86

Table S4. Exploratory factor analysis: perceived privacy of information—Bartlett's test for sphericity.

Chi-squared	p-value	df
3,487.02	<.001	6

Table S5. Exploratory factor analysis: perceived privacy of information—Eigenvalues.

Factors	Eigenvalues
1	2.70
2	0.60
3	0.43
4	0.27

Table S6. Exploratory factor analysis: perceived privacy of information—uniqueness and factor loadings (Varimax).

Variables	Uniqueness	Loadings F1
Perceived privacy of current location data	.43	.75
Perceived privacy of travel behavior data	.21	.89
Perceived privacy of physical activity data	.42	.76
Perceived privacy of personal health data	.63	.61

Table S7. Exploratory factor analysis: trust in institutions—Kaiser-Meyer-Olkin (KMO) test.

Variable		MSA
Overall		.83
	Trust in pollsters and market research agencies	.87
	Trust in scientific researchers	.81
	Trust in government institutions	.81
	Trust in official statistical institutes	.78
	Trust in companies that make apps	.88
	Trust in online stores	.88
	Trust in social media companies	.83
	Trust in technology companies	.82

Table S8. Exploratory factor analysis: trust in institutions—Bartlett's test for sphericity.

Chi-squared	p-value	df
8,302.44	<.001	28

Table S9. Exploratory factor analysis: trust in institutions—Eigenvalues.

Factors	Eigenvalues
1	3.85
2	1.81
3	0.64
4	0.42
5	0.41
6	0.34
7	0.27
8	0.26

Table S10. Exploratory factor analysis: trust in institutions—uniqueness and factor loadings (Varimax).

Variables	Uniqueness	Loadings F1	Loadings F2
Trust in pollsters and market research agencies	.65	.27	.52
Trust in scientific researchers	.44	<.10	.75
Trust in government institutions	.36	.19	.78
Trust in official statistical institutes	.21	.15	.88
Trust in companies that make apps	.40	.72	.29
Trust in online stores	.41	.75	.19
Trust in social media companies	.34	.81	<.10
Trust in technology companies	.27	.84	.16

Table S11. Exploratory factor analysis: limitation by health in activities—Kaiser-Meyer-Olkin (KMO) test.

Variable		MSA
Overall		.76
	Limited by health in daily activities	.79
	Limited by health in social activities	.73
	Limited by health in work	.75

Table S12. Exploratory factor analysis: limitation by health in activities—Bartlett's test for sphericity.

Chi-squared	p-value	df
4,396.41	<.001	3

Table S13. Exploratory factor analysis: limitation by health in activities—Eigenvalues.

Factors	Eigenvalues
1	2.56
2	0.24
3	0.19

Table S14. Exploratory factor analysis: limitation by health in activities—uniqueness and factor loadings (Varimax).

Variables	Uniqueness	Loadings F1
Limited by health in daily activities	.26	.86
Limited by health in social activities	.18	.91
Limited by health in work	.22	.88

Table S15. Exploratory factor analysis: difficulties with tasks—Kaiser-Meyer-Olkin (KMO) test.

Variable		MSA
Overall		.92
	Walking 100 meters	.95
	Sitting for around two hours	.94
	Getting up from chair	.94
	Walking several stairs without resting	.90
	Walking up staircase without resting	.90
	Crouching, kneeling, crawling	.94
	Reaching above shoulder height	.95
	Moving large objects	.91
	Lifting or carrying 5 kilos	.90
	Picking up small coin from table	.97

Table S16. Exploratory factor analysis: difficulties with tasks—Bartlett's test for sphericity.

Chi-squared	p-value	df
12,323.65	<.001	45

Table S17. Exploratory factor analysis: difficulties with tasks—Eigenvalues.

Factors	Eigenvalues
1	5.67

2	0.86
3	0.73
4	0.66
5	0.56
6	0.41
7	0.38
8	0.33
9	0.21
10	0.20

Table S18. Exploratory factor analysis: difficulties with tasks—uniqueness and factor loadings (Varimax).

Variables	Uniqueness	Loadings F1
Walking 100 meters	.45	.74
Sitting for around two hours	.75	.50
Getting up from chair	.51	.70
Walking several stairs without resting	.32	.82
Walking up staircase without resting	.31	.83
Crouching, kneeling, crawling	.42	.76
Reaching above shoulder height	.57	.66
Moving large objects	.34	.81
Lifting or carrying 5 kilos	.35	.81
Picking up small coin from table	.75	.50
