# Supplementary material for: Data Donation as a Method to Measure Physical Activity in Older Adults: Cross-Sectional Web Survey Assessing Consent Rates, Donation Success, and Bias
Source: J Med Internet Res. 2025 Sep 26;27:e69799. doi: 10.2196/69799 (PMC12514404; doi:10.2196/69799)
Supplement: Multimedia Appendix 3 [file jmir_v27i1e69799_app3.pdf]

## Multimedia Appendix 3

Table S1. Average marginal effects (AME), 95% confidence intervals (CI), and p-values from logistic regression model predicting completing survey and being included in the analytical sample (model 0).

| Variables | | AME (95% CI) | *P*-value |
|---|---|---|---|
| **Gender** | | | |
| | Female | Ref. | Ref. |
| | Male | 0.02 (-0.01 – 0.04) | .254 |
| **Age** | | | |
| | 50-54 years | Ref. | Ref. |
| | 55-59 years | -0.02 (-0.07 – 0.03) | .456 |
| | 60-64 years | -0.05 (-0.10 – -0.01) | .027 |
| | 65-69 years | -0.05 (-0.11 – 0.01) | .099 |
| | 70-74 years | -0.08 (-0.14 – -0.02) | .006 |
| | 75-79 years | -0.04 (-0.11 – 0.03) | .257 |
| | 80 years and older | -0.05 (-0.12 – 0.01) | .121 |
| **HH size** | | | |
| | Single-person HH | Ref. | Ref. |
| | Two-person HH | 0.00 (-0.03 – 0.03) | .949 |
| | Three- and more person HH | 0.03 (-0.01 – 0.07) | .166 |
| **Urbanicity** | | | |
| | Not urban | Ref. | Ref. |
| | Little urban | 0.01 (-0.03 – 0.05) | .582 |
| | Moderately urban | 0.02 (-0.02 – 0.06) | .405 |
| | Strongly urban | 0.01 (-0.03 – 0.05) | .533 |
| | Very strongly urban | 0.03 (-0.01 – 0.08) | .160 |
| **Employment status** | | | |
| | Employed for pay | Ref. | Ref. |
| | Unpaid work, incl. housework | -0.05 (-0.10 – 0.00) | .062 |
| | Unemployed, retired, or disabled | -0.06 (-0.11 – -0.02) | .004 |
| **Monthly personal net income** | | | |
| | Up to EUR 1,000 | Ref. | Ref. |
| | EUR 1,001 – EUR 1,500 | 0.05 (-0.02 – 0.11) | .144 |
| | EUR 1,501 – EUR 2,000 | -0.01 (-0.07 – 0.04) | .656 |
| | EUR 2,001 – EUR 2,500 | -0.02 (-0.08 – 0.03) | .437 |
| | EUR 2,501 – EUR 3,000 | -0.02 (-0.08 – 0.04) | .543 |
| | More than EUR 3,000 | -0.03 (-0.09 – 0.02) | .256 |
| | No income/NA | 0.00 (-0.06 – 0.07) | .899 |
| **Educational attainment** | | | |
| | Low | Ref. | Ref. |
| | Medium | -0.02 (-0.06 – 0.01) | .179 |
| | High | -0.00 (-0.04 – 0.03) | .889 |
| n | | 2,336[a] | |
| AIC | | 1,590.2 | |
| McFadden Pseudo $R^2$ | | .045 | |

[a]Out of the 2,345 LISS panel members who were invited to the survey, 9 had to be dropped from the analysis due to missing data in the covariates.

Table S2. Average marginal effects (AME), 95% confidence intervals (CI), and p-values from weighted logistic regression model predicting owning an iPhone or Android phone adjusted for nonresponse to the survey among LISS panel members (model 1a).

| Variables | | AME (95% CI) | P-value |
|---|---|---|---|
| **Gender** | | | |
| | Female | Ref. | Ref. |
| | Male | -0.04 (-0.07 – -0.02) | .001 |
| **Age** | | | |
| | 50-54 years | Ref. | Ref. |
| | 55-59 years | -0.01 (-0.06 – 0.04) | .651 |
| | 60-64 years | -0.01 (-0.06 – 0.03) | .501 |
| | 65-69 years | -0.04 (-0.09 – 0.01) | .106 |
| | 70-74 years | -0.05 (-0.10 – -0.00) | .040 |
| | 75-79 years | -0.10 (-0.16 – -0.03) | .002 |
| | 80 years and older | -0.20 (-0.28 – -0.12) | <.001 |
| **HH size** | | | |
| | Single-person HH | Ref. | Ref. |
| | Two-person HH | 0.02 (-0.01 – 0.05) | .112 |
| | Three- and more person HH | -0.01 (-0.07 – 0.05) | .794 |
| **Urbanicity** | | | |
| | Not urban | Ref. | Ref. |
| | Little urban | 0.02 (-0.02 – 0.06) | .340 |
| | Moderately urban | 0.00 (-0.04 – 0.04) | .907 |
| | Strongly urban | -0.01 (-0.04 – 0.03) | .773 |
| | Very strongly urban | 0.01 (-0.03 – 0.05) | .774 |
| **Employment status** | | | |
| | Employed for pay | Ref. | Ref. |
| | Unpaid work, incl. housework | -0.00 (-0.06 – 0.05) | .871 |
| | Unemployed, retired, or disabled | 0.01 (-0.04 – 0.06) | .725 |
| **Monthly personal net income** | | | |
| | Up to EUR 1,000 | Ref. | Ref. |
| | EUR 1,001 – EUR 1,500 | 0.03 (-0.03 – 0.08) | .331 |
| | EUR 1,501 – EUR 2,000 | 0.07 (0.02 – 0.12) | .007 |
| | EUR 2,001 – EUR 2,500 | 0.09 (0.03 – 0.14) | .001 |
| | EUR 2,501 – EUR 3,000 | 0.07 (0.01 – 0.13) | .015 |
| | More than EUR 3,000 | 0.10 (0.04 – 0.15) | <.001 |
| | No income/NA | -0.02 (-0.09 – 0.05) | .623 |
| **Educational attainment** | | | |
| | Low | Ref. | Ref. |
| | Medium | 0.04 (0.01 – 0.07) | .013 |
| | High | 0.07 (0.04 – 0.10) | <.001 |
| **General privacy concerns** | | | |
| | Not at all concerned | Ref. | Ref. |
| | Not very concerned | 0.07 (0.02 – 0.12) | .009 |
| | A little concerned | 0.09 (0.05 – 0.14) | <.001 |
| | Very concerned | 0.05 (-0.01 – 0.12) | .095 |
| **Perceived privacy of information** | | -0.00 (-0.01 – 0.01) | .655 |
| **Trust in government and research institutions** | | -0.01 (-0.03 – 0.01) | .285 |
| **Trust in technology companies** | | 0.01 (-0.00 – 0.03) | .138 |

| | | | |
|---|---|---|---|
| **Self-rated health** | | | |
| | Moderate/Bad | Ref. | Ref. |
| | Good | -0.02 (-0.05 – 0.01) | .186 |
| | Excellent/Very good | -0.01 (-0.06 – 0.03) | .526 |
| **Chronic illness** | | | |
| | No | Ref. | Ref. |
| | Yes | -0.03 (-0.06 – -0.01) | .013 |
| **BMI** | | | |
| | Underweight/Healthy weight | Ref. | Ref. |
| | Overweight | 0.02 (-0.01 – 0.04) | .161 |
| | Obesity | 0.01 (-0.02 – 0.05) | .424 |
| **Limited in activities by health** | | 0.01 (-0.01 – 0.03) | .343 |
| **Difficulties with tasks** | | -0.04 (-0.07 – -0.02) | .001 |
| **No. days with moderate physical activity** | | 0.00 (-0.00 – 0.01) | .605 |
| **No. days with strenuous physical activity** | | 0.00 (-0.00 – 0.01) | .349 |
| **No. days walking** | | 0.00 (-0.00 – 0.01) | .306 |
| **No. days running** | | -0.01 (-0.03 – 0.01) | .318 |
| **No. days biking** | | -0.01 (-0.01 – 0.00) | .056 |
| **Time sedentary in h** | | -0.00 (-0.00 – 0.00) | .877 |
| **Spending time outdoors yesterday** | | | |
| | No | Ref. | Ref. |
| | Yes | -0.01 (-0.04 – 0.02) | .417 |
| n | | 2,020[a] | |
| AIC | | 1,334.3 | |
| McFadden Pseudo $R^2$ | | .161 | |

[a]Out of the 2,086 respondents in the survey, 66 had to be dropped from the analysis due to missing data in the covariates.
