# Supplementary material for: Data Donation as a Method to Measure Physical Activity in Older Adults: Cross-Sectional Web Survey Assessing Consent Rates, Donation Success, and Bias
Source: J Med Internet Res. 2025 Sep 26;27:e69799. doi: 10.2196/69799 (PMC12514404; doi:10.2196/69799)
Supplement: Multimedia Appendix 4 [file jmir_v27i1e69799_app4.pdf]

Multimedia Appendix 4

Table S1. Comparison of results of full logistic regression model predicting owning an iPhone or Android phone (model 1) with more parsimonious models.

		Full model (M1)		Sociodemo., privacy, & trust		Sociodemo. & health		Sociodemo. & PA	
Variables		AME (95% CI)	P-val.	AME (95% CI)	P-val.	AME (95% CI)	P-val.	AME (95% CI)	P-val.
**Gender**									
	Female	Ref.	Ref.	Ref.	Ref.	Ref.	Ref.	Ref.	Ref.
	Male	-0.05 (-0.08 – -0.02)	.001	-0.04 (-0.07 – -0.01)	.003	-0.06 (-0.09 – -0.03)	<.001	-0.04 (-0.07 – -0.02)	.002
**Age**									
	50-54 years	Ref.	Ref.	Ref.	Ref.	Ref.	Ref.	Ref.	Ref.
	55-59 years	-0.01 (-0.05 – 0.03)	.649	-0.01 (-0.04 – 0.03)	.635	-0.01 (-0.04 – 0.03)	.666	-0.01 (-0.05 – 0.03)	.650
	60-64 years	-0.02 (-0.06 – 0.02)	.287	-0.02 (-0.06 – 0.01)	.188	-0.02 (-0.06 – 0.01)	.243	-0.02 (-0.06 – 0.01)	.229
	65-69 years	-0.03 (-0.07 – 0.02)	.218	-0.03 (-0.07 – 0.01)	.143	-0.03 (-0.07 – 0.01)	.209	-0.03 (-0.07 – 0.01)	.185
	70-74 years	-0.05 (-0.09 – 0.00)	.063	-0.04 (-0.09 – -0.00)	.048	-0.05 (-0.10 – -0.00)	.042	-0.04 (-0.09 – 0.00)	.068
	75-79 years	-0.08 (-0.14 – -0.03)	.004	-0.10 (-0.15 – -0.04)	.001	-0.09 (-0.15 – -0.03)	.002	-0.09 (-0.15 – -0.04)	.001
	80 years and older	-0.19 (-0.27 – -0.12)	<.001	-0.21 (-0.28 – -0.13)	<.001	-0.21 (-0.29 – -0.13)	<.001	-0.22 (-0.30 – -0.14)	<.001
**HH size**									
	Single-person HH	Ref.	Ref.	Ref.	Ref.	Ref.	Ref.	Ref.	Ref.
	Two-person HH	0.03 (-0.00 – 0.06)	.101	0.02 (-0.01 – 0.06)	.110	0.03 (-0.01 – 0.06)	.106	0.02 (-0.01 – 0.05)	.130
	Three- and more person HH	0.01 (-0.04 – 0.06)	.594	0.01 (-0.03 – 0.06)	.557	0.01 (-0.04 – 0.06)	.621	0.01 (-0.04 – 0.06)	.662
**Urbanicity**									
	Not urban	Ref.	Ref.	Ref.	Ref.	Ref.	Ref.	Ref.	Ref.
	Little urban	0.02 (-0.02 – 0.06)	.371	0.02 (-0.03 – 0.06)	.466	0.02 (-0.02 – 0.06)	.343	0.02 (-0.02 – 0.06)	.318
	Moderately urban	0.01 (-0.03 – 0.06)	.582	0.00 (-0.04 – 0.04)	.998	0.01 (-0.03 – 0.05)	.678	0.01 (-0.04 – 0.05)	.782
	Strongly urban	0.01 (-0.03 – 0.05)	.524	0.00 (-0.04 – 0.04)	.978	0.01 (-0.03 – 0.05)	.522	0.01 (-0.03 – 0.05)	.688
	Very strongly urban	0.01 (-0.04 – 0.06)	.667	0.00 (-0.04 – 0.05)	.921	0.01 (-0.03 – 0.06)	.644	0.01 (-0.04 – 0.05)	.820
**Employment status**									
	Employed for pay	Ref.	Ref.	Ref.	Ref.	Ref.	Ref.	Ref.	Ref.
	Unpaid work, incl. housework	-0.02 (-0.07 – 0.04)	.533	-0.02 (-0.07 – 0.02)	.336	-0.01 (-0.07 – 0.04)	.598	-0.02 (-0.07 – 0.03)	.446
	Unemployed, retired, or disabled	-0.00 (-0.05 – 0.04)	.932	-0.02 (-0.06 – 0.02)	.398	-0.00 (-0.04 – 0.04)	.995	-0.02 (-0.06 – 0.03)	.435
**Monthly personal net income**									
	Up to EUR 1,000	Ref.	Ref.	Ref.	Ref.	Ref.	Ref.	Ref.	Ref.
	EUR 1,001 – EUR 1,500	0.04 (-0.02 – 0.10)	.164	0.06 (-0.00 – 0.11)	.059	0.04 (-0.02 – 0.10)	.217	0.04 (-0.01 – 0.10)	.137
	EUR 1,501 – EUR 2,000	0.07 (0.02 – 0.13)	.012	0.09 (0.03 – 0.15)	.002	0.07 (0.02 – 0.13)	.012	0.08 (0.03 – 0.14)	.004
	EUR 2,001 – EUR 2,500	0.08 (0.03 – 0.14)	.005	0.10 (0.04 – 0.16)	.001	0.09 (0.03 – 0.15)	.003	0.09 (0.04 – 0.15)	.002
	EUR 2,501 – EUR 3,000	0.06 (0.00 – 0.13)	.046	0.08 (0.02 – 0.15)	.013	0.06 (-0.00 – 0.13)	.056	0.07 (0.01 – 0.14)	.031

	More than EUR 3,000	0.11 (0.05 – 0.17)	<.001	0.13 (0.07 – 0.19)	<.001	0.11 (0.06 – 0.17)	<.001	0.12 (0.06 – 0.18)	<.001
	No income/NA	0.00 (-0.07 – 0.07)	.994	0.02 (-0.05 – 0.09)	.576	0.01 (-0.07 – 0.08)	.865	0.01 (-0.06 – 0.08)	.752
**Educational attainment**									
	Low	Ref.	Ref.	Ref.	Ref.	Ref.	Ref.	Ref.	Ref.
	Medium	0.03 (-0.00 – 0.07)	.061	0.03 (0.00 – 0.07)	.044	0.03 (-0.00 – 0.06)	.066	0.03 (0.00 – 0.07)	.042
	High	0.06 (0.03 – 0.10)	<.001	0.06 (0.03 – 0.10)	<.001	0.06 (0.03 – 0.09)	<.001	0.07 (0.03 – 0.10)	<.001
**General privacy concerns**									
	Not at all concerned	Ref.	Ref.	Ref.	Ref.				
	Not very concerned	0.06 (0.01 – 0.11)	.027	0.06 (0.00 – 0.11)	.035				
	A little concerned	0.08 (0.03 – 0.14)	.001	0.09 (0.04 – 0.14)	.001				
	Very concerned	0.05 (-0.01 – 0.12)	.121	0.06 (-0.01 – 0.12)	.083				
**Perceived privacy of information**		0.00 (-0.01 – 0.02)	.503	0.01 (-0.01 – 0.02)	.294				
**Trust in government and research institutions**		0.00 (-0.02 – 0.02)	.901	-0.00 (-0.02 – 0.02)	.986				
**Trust in technology companies**		0.01 (-0.01 – 0.03)	.282	0.01 (-0.01 – 0.03)	.311				
**Self-rated health**									
	Moderate/Bad	Ref.	Ref.			Ref.	Ref.		
	Good	-0.01 (-0.04 – 0.02)	.544			-0.01 (-0.05 – 0.02)	.395		
	Excellent/Very good	-0.01 (-0.06 – 0.04)	.792			-0.01 (-0.06 – 0.04)	.616		
**Chronic illness**									
	No	Ref.	Ref.			Ref.	Ref.		
	Yes	-0.02 (-0.05 – 0.01)	.134			-0.02 (-0.05 – 0.01)	.267		
**BMI**									
	Underweight/Healthy weight	Ref.	Ref.			Ref.	Ref.		
	Overweight	0.01 (-0.01 – 0.04)	.329			0.01 (-0.01 – 0.04)	.301		
	Obesity	0.00 (-0.03 – 0.04)	.811			0.00 (-0.03 – 0.04)	.832		
**Limited in activities by health**		0.01 (-0.01 – 0.03)	.343			0.01 (-0.01 – 0.02)	.409		
**Difficulties with tasks**		-0.04 (-0.07 – -0.02)	.001			-0.05 (-0.07 – -0.02)	<.001		
**No. days with moderate physical activity**		0.00 (-0.00 – 0.01)	.605					0.00 (-0.00 – 0.01)	.354
**No. days with strenuous physical activity**		0.00 (-0.00 – 0.01)	.349					0.00 (-0.00 – 0.01)	.405
**No. days walking**		0.00 (-0.00 – 0.01)	.306					0.00 (-0.00 – 0.01)	.156
**No. days running**		-0.01 (-0.03 – 0.01)	.318					-0.01 (-0.03 – 0.01)	.453
**No. days biking**		-0.01 (-0.01 – 0.00)	.056					-0.01 (-0.01 – 0.00)	.050
**Time sedentary in h**		0.00 (-0.00 – 0.00)	.865					-0.00 (-0.01 – 0.00)	.462
**Spending time outdoors yesterday**									
	No	Ref.	Ref.					Ref.	Ref.
	Yes	-0.01 (-0.04 – 0.02)	.587					-0.01 (-0.04 – 0.02)	.621
n		2,020[a]		2,078[b]		2,022[c]		2,080[d]	

AIC	1,117.8		1,146.2		1,118.2		1,162.1	
McFadden Pseudo R^2	.174		.161		.156		.153	

[a]Out of the 2,086 respondents in the survey, 66 had to be dropped from the analysis due to missing data in the covariates.
[b]Out of the 2,086 respondents in the survey, 8 had to be dropped from the analysis due to missing data in the covariates.
[c]Out of the 2,086 respondents in the survey, 64 had to be dropped from the analysis due to missing data in the covariates.
[d]Out of the 2,086 respondents in the survey, 6 had to be dropped from the analysis due to missing data in the covariates.

Table S2. Comparison of results of full logistic regression model predicting willingness to donate PA data conditional on being asked (model 2) with more parsimonious models.

		Full model (M2)		Sociodemo., privacy, & trust		Sociodemo. & health		Sociodemo. & PA		Sociodemo. & others	
Variables		AME (95% CI)	P-val.		P-val.	AME (95% CI)	P-val.	AME (95% CI)	P-val.	AME (95% CI)	P-val.
**Gender**											
	Female	Ref.	Ref.	Ref.	Ref.	Ref.	Ref.	Ref.	Ref.	Ref.	Ref.
	Male	0.07 (0.02 – 0.11)	.006	0.04 (-0.01 – 0.08)	.119	0.03 (-0.02 – 0.08)	.190	0.03 (-0.01 – 0.08)	.143	0.06 (0.02 – 0.11)	.005
**Age**											
	50-54 years	Ref.	Ref.	Ref.	Ref.	Ref.	Ref.	Ref.	Ref.	Ref.	Ref.
	55-59 years	0.00 (-0.07 – 0.08)	.967	-0.01 (-0.09 – 0.07)	.883	-0.01 (-0.09 – 0.07)	.853	-0.00 (-0.08 – 0.08)	.923	0.01 (-0.06 – 0.09)	.746
	60-64 years	-0.07 (-0.15 – 0.01)	.087	-0.10 (-0.18 – -0.02)	.015	-0.08 (-0.17 – -0.00)	.043	-0.09 (-0.17 – -0.01)	.031	-0.04 (-0.12 – 0.03)	.253
	65-69 years	-0.09 (-0.18 – 0.00)	.051	-0.14 (-0.23 – -0.04)	.004	-0.12 (-0.22 – -0.03)	.013	-0.12 (-0.22 – -0.03)	.010	-0.08 (-0.17 – 0.01)	.078
	70-74 years	-0.08 (-0.18 – 0.02)	.097	-0.15 (-0.25 – -0.05)	.003	-0.13 (-0.23 – -0.02)	.017	-0.14 (-0.24 – -0.04)	.008	-0.08 (-0.17 – 0.02)	.122
	75-79 years	-0.14 (-0.24 – -0.03)	.011	-0.22 (-0.32 – -0.12)	<.001	-0.20 (-0.30 – -0.10)	<.001	-0.21 (-0.31 – -0.11)	<.001	-0.12 (-0.22 – -0.02)	.021
	80 years and older	-0.14 (-0.26 – -0.02)	.020	-0.25 (-0.35 – -0.15)	<.001	-0.24 (-0.34 – -0.13)	<.001	-0.23 (-0.34 – -0.13)	<.001	-0.11 (-0.23 – 0.00)	.058
**HH size**											
	Single-person HH	Ref.	Ref.	Ref.	Ref.	Ref.	Ref.	Ref.	Ref.	Ref.	Ref.
	Two-person HH	-0.07 (-0.12 – -0.01)	.015	-0.05 (-0.10 – 0.01)	.093	-0.05 (-0.10 – 0.00)	.074	-0.05 (-0.10 – 0.01)	.085	-0.06 (-0.11 – -0.01)	.021
	Three- and more person HH	-0.10 (-0.16 – -0.03)	.004	-0.09 (-0.15 – -0.02)	.011	-0.07 (-0.14 – 0.00)	.061	-0.08 (-0.14 – -0.01)	.026	-0.09 (-0.16 – -0.03)	.005
**Urbanicity**											
	Not urban	Ref.	Ref.	Ref.	Ref.	Ref.	Ref.	Ref.	Ref.	Ref.	Ref.
	Little urban	-0.04 (-0.12 – 0.03)	.245	-0.04 (-0.12 – 0.03)	.235	-0.05 (-0.12 – 0.03)	.240	-0.04 (-0.12 – 0.03)	.244	-0.05 (-0.12 – 0.02)	.185
	Moderately urban	-0.08 (-0.15 – -0.00)	.036	-0.08 (-0.15 – -0.01)	.033	-0.07 (-0.14 – 0.01)	.073	-0.08 (-0.15 – -0.00)	.041	-0.09 (-0.16 – -0.02)	.015
	Strongly urban	-0.11 (-0.18 – -0.05)	.001	-0.10 (-0.16 – -0.03)	.005	-0.10 (-0.17 – -0.03)	.005	-0.10 (-0.17 – -0.03)	.004	-0.12 (-0.19 – -0.05)	<.001
	Very strongly urban	-0.10 (-0.18 – -0.03)	.006	-0.10 (-0.17 – -0.02)	.010	-0.09 (-0.17 – -0.02)	.016	-0.10 (-0.18 – -0.03)	.008	-0.11 (-0.18 – -0.03)	.004
**Employment status**											
	Employed for pay	Ref.	Ref.	Ref.	Ref.	Ref.	Ref.	Ref.	Ref.	Ref.	Ref.
	Unpaid work, incl. housework	-0.02 (-0.10 – 0.07)	.692	-0.04 (-0.12 – 0.05)	.374	-0.04 (-0.13 – 0.04)	.335	-0.03 (-0.11 – 0.06)	.513	-0.01 (-0.09 – 0.07)	.763
	Unemployed, retired, or disabled	0.03 (-0.03 – 0.10)	.315	0.03 (-0.04 – 0.09)	.462	0.01 (-0.06 – 0.08)	.750	0.03 (-0.04 – 0.10)	.355	0.05 (-0.01 – 0.12)	.116
**Monthly personal net income**											
	Up to EUR 1,000	Ref.	Ref.	Ref.	Ref.	Ref.	Ref.	Ref.	Ref.	Ref.	Ref.

	EUR 1,001 – EUR 1,500	-0.01 (-0.10 – 0.08)	.834	-0.01 (-0.09 – 0.08)	.905	0.01 (-0.08 – 0.10)	.798	0.01 (-0.08 – 0.10)	.778	-0.01 (-0.10 – 0.08)	.787
	EUR 1,501 – EUR 2,000	0.02 (-0.07 – 0.11)	.718	0.05 (-0.04 – 0.14)	.244	0.07 (-0.02 – 0.16)	.129	0.06 (-0.02 – 0.15)	.161	0.03 (-0.06 – 0.12)	.569
	EUR 2,001 – EUR 2,500	-0.02 (-0.11 – 0.07)	.684	0.03 (-0.06 – 0.12)	.540	0.04 (-0.05 – 0.12)	.429	0.03 (-0.05 – 0.12)	.436	-0.01 (-0.10 – 0.08)	.832
	EUR 2,501 – EUR 3,000	0.03 (-0.06 – 0.13)	.479	0.09 (-0.01 – 0.18)	.068	0.11 (0.02 – 0.21)	.022	0.11 (0.01 – 0.20)	.028	0.05 (-0.04 – 0.15)	.277
	More than EUR 3,000	0.05 (-0.05 – 0.15)	.313	0.13 (0.04 – 0.22)	.006	0.16 (0.06 – 0.25)	.001	0.15 (0.05 – 0.24)	.002	0.06 (-0.04 – 0.15)	.230
	No income/NA	-0.07 (-0.17 – 0.03)	.154	-0.03 (-0.12 – 0.07)	.566	-0.05 (-0.14 – 0.05)	.315	-0.04 (-0.13 – 0.06)	.441	-0.08 (-0.17 – 0.02)	.102
**Educational attainment**											
	Low	Ref.	Ref.	Ref.	Ref.	Ref.	Ref.	Ref.	Ref.	Ref.	Ref.
	Medium	0.05 (-0.00 – 0.11)	.057	0.07 (0.01 – 0.12)	.016	0.07 (0.01 – 0.12)	.019	0.06 (0.01 – 0.12)	.022	0.05 (0.00 – 0.11)	.048
	High	0.10 (0.04 – 0.16)	.001	0.12 (0.06 – 0.18)	<.001	0.11 (0.05 – 0.17)	<.001	0.11 (0.05 – 0.17)	<.001	0.10 (0.04 – 0.16)	.001
**General privacy concerns**											
	Not at all concerned	Ref.	Ref.	Ref.	Ref.						
	Not very concerned	-0.03 (-0.11 – 0.06)	.503	-0.04 (-0.12 – 0.05)	.384						
	A little concerned	-0.06 (-0.14 – 0.03)	.186	-0.05 (-0.13 – 0.03)	.244						
	Very concerned	-0.02 (-0.13 – 0.09)	.711	-0.04 (-0.15 – 0.06)	.426						
**Perceived privacy of information**		-0.04 (-0.06 – -0.02)	<.001	-0.04 (-0.06 – -0.02)	<.001						
**Trust in government and research institutions**		0.05 (0.01 – 0.09)	.006	0.06 (0.03 – 0.10)	.001						
**Trust in technology companies**		0.01 (-0.02 – 0.05)	.530	0.03 (-0.01 – 0.06)	.154						
**Self-rated health**											
	Moderate/Bad	Ref.	Ref.			Ref.	Ref.				
	Good	-0.06 (-0.13 – 0.00)	.056			-0.05 (-0.12 – 0.02)	.148				
	Excellent/Very good	-0.05 (-0.13 – 0.03)	.251			-0.02 (-0.11 – 0.07)	.702				
**Chronic illness**											
	No	Ref.	Ref.			Ref.	Ref.				
	Yes	0.06 (0.01 – 0.11)	.011			0.07 (0.01 – 0.12)	.011				
**BMI**											
	Underweight/Healthy weight	Ref.	Ref.			Ref.	Ref.				
	Overweight	-0.04 (-0.08 – 0.01)	.105			-0.03 (-0.08 – 0.02)	.189				
	Obesity	-0.02 (-0.08 – 0.04)	.421			-0.01 (-0.07 – 0.05)	.716				

**Limited in activities by health**	-0.02 (-0.05 – 0.01)	.155			-0.03 (-0.06 – 0.00)	.081				
**Difficulties with tasks**	0.00 (-0.06 – 0.06)	.980			-0.00 (-0.06 – 0.05)	.906				
**No. days with moderate physical activity**	-0.00 (-0.01 – 0.01)	.956					-0.00 (-0.01 – 0.01)	.493		
**No. days with strenuous physical activity**	0.01 (-0.00 – 0.02)	.181					0.01 (-0.00 – 0.02)	.066		
**No. days walking**	-0.00 (-0.01 – 0.01)	.592					0.00 (-0.01 – 0.01)	.463		
**No. days running**	-0.02 (-0.05 – 0.02)	.271					-0.02 (-0.05 – 0.02)	.292		
**No. days biking**	0.01 (-0.00 – 0.02)	.231					0.01 (-0.00 – 0.02)	.246		
**Time sedentary in h**	0.00 (-0.00 – 0.01)	.278					0.01 (-0.00 – 0.01)	.142		
**Spending time outdoors yesterday**										
No	Ref.	Ref.					Ref.	Ref.		
Yes	0.04 (-0.02 – 0.10)	.157					0.06 (-0.00 – 0.12)	.060		
**No. of smartphone activities**	0.03 (0.02 – 0.04)	<.001							0.03 (0.02 – 0.04)	<.001
**Type of data requested**										
Apple Health	Ref.	Ref.							Ref.	Ref.
Google Location History	-0.14 (-0.18 – -0.09)	<.001							-0.13 (-0.17 – -0.08)	<.001
Samsung Health	-0.07 (-0.14 – 0.00)	.061							-0.06 (-0.14 – 0.01)	.093
**Data example shown**										
No	Ref.	Ref.							Ref.	Ref.
Yes	-0.05 (-0.09 – -0.01)	.023							-0.05 (-0.09 – -0.01)	.022
n	1,830[a]		1,878[b]		1,830[c]		1,878[d]		1,878[e]	
AIC	2,097.5		2,241.5		2,219.7		2,269.2		2,154.5	
McFadden Pseudo R^2	.134		.078		.066		.064		.110	

[a]Out of the 1,883 respondents who were asked the willingness question, 53 had to be dropped from the analysis due to missing data in the covariates.
[b]Out of the 1,883 respondents who were asked the willingness question, 5 had to be dropped from the analysis due to missing data in the covariates.
[c]Out of the 1,883 respondents who were asked the willingness question, 53 had to be dropped from the analysis due to missing data in the covariates.
[d]Out of the 1,883 respondents who were asked the willingness question, 5 had to be dropped from the analysis due to missing data in the covariates.
[e]Out of the 1,883 respondents who were asked the willingness question, 5 had to be dropped from the analysis due to missing data in the covariates.

Table S3. Comparison of results of full logistic regression model predicting starting to donate PA data conditional on willingness (model 3) with more parsimonious models.

		Full model (M3)		Sociodemo., privacy, & trust		Sociodemo. & health		Sociodemo. & PA		Sociodemo. & others	
Variables		AME (95% CI)	P-val.		P-val.	AME (95% CI)	P-val.	AME (95% CI)	P-val.	AME (95% CI)	P-val.
Gender											
	Female	Ref.	Ref.	Ref.	Ref.	Ref.	Ref.	Ref.	Ref.	Ref.	Ref.
	Male	0.04 (-0.04 – 0.12)	.315	-0.01 (-0.09 – 0.07)	.834	-0.01 (-0.10 – 0.07)	.773	-0.02 (-0.10 – 0.06)	.645	0.03 (-0.05 – 0.11)	.493
Age											
	50-54 years	Ref.	Ref.	Ref.	Ref.	Ref.	Ref.	Ref.	Ref.	Ref.	Ref.
	55-59 years	-0.08 (-0.19 – 0.03)	.161	-0.11 (-0.22 – 0.00)	.056	-0.12 (-0.23 – -0.00)	.045	-0.12 (-0.24 – -0.00)	.044	-0.11 (-0.22 – 0.01)	.07
	60-64 years	-0.10 (-0.22 – 0.02)	.098	-0.14 (-0.25 – -0.02)	.024	-0.12 (-0.24 – -0.00)	.042	-0.12 (-0.24 – -0.00)	.043	-0.09 (-0.21 – 0.03)	.159
	65-69 years	-0.20 (-0.34 – -0.06)	.005	-0.23 (-0.36 – -0.09)	.001	-0.24 (-0.38 – -0.09)	.001	-0.22 (-0.37 – -0.08)	.002	-0.18 (-0.31 – -0.04)	.014
	70-74 years	-0.19 (-0.35 – -0.04)	.015	-0.23 (-0.39 – -0.08)	.003	-0.24 (-0.40 – -0.09)	.002	-0.23 (-0.39 – -0.07)	.004	-0.20 (-0.36 – -0.04)	.013
	75-79 years	-0.41 (-0.59 – -0.24)	<.001	-0.43 (-0.60 – -0.26)	<.001	-0.46 (-0.63 – -0.29)	<.001	-0.46 (-0.63 – -0.29)	<.001	-0.42 (-0.60 – -0.25)	<.001
	80 years and older	-0.44 (-0.65 – -0.23)	<.001	-0.53 (-0.71 – -0.34)	<.001	-0.58 (-0.75 – -0.42)	<.001	-0.59 (-0.75 – -0.43)	<.001	-0.51 (-0.70 – -0.31)	<.001
HH size											
	Single-person HH	Ref.	Ref.	Ref.	Ref.	Ref.	Ref.	Ref.	Ref.	Ref.	Ref.
	Two-person HH	0.06 (-0.03 – 0.15)	.173	0.12 (0.03 – 0.21)	.012	0.09 (-0.01 – 0.19)	.073	0.11 (0.01 – 0.20)	.031	0.06 (-0.03 – 0.16)	.179
	Three- and more person HH	-0.04 (-0.15 – 0.08)	.520	0.01 (-0.10 – 0.13)	.817	-0.05 (-0.17 – 0.07)	.460	-0.03 (-0.15 – 0.09)	.629	-0.06 (-0.18 – 0.05)	.289
Urbanicity											
	Not urban	Ref.	Ref.	Ref.	Ref.	Ref.	Ref.	Ref.	Ref.	Ref.	Ref.
	Little urban	-0.01 (-0.13 – 0.10)	.808	-0.02 (-0.14 – 0.10)	.708	0.01 (-0.11 – 0.13)	.854	-0.01 (-0.13 – 0.11)	.886	-0.01 (-0.13 – 0.11)	.839
	Moderately urban	0.00 (-0.11 – 0.12)	.956	-0.02 (-0.14 – 0.10)	.708	-0.00 (-0.13 – 0.12)	.950	-0.02 (-0.15 – 0.10)	.697	-0.03 (-0.15 – 0.09)	.622
	Strongly urban	-0.03 (-0.14 – 0.08)	.578	-0.01 (-0.12 – 0.10)	.868	-0.02 (-0.13 – 0.10)	.768	-0.02 (-0.13 – 0.10)	.755	-0.04 (-0.15 – 0.07)	.462
	Very strongly urban	-0.07 (-0.20 – 0.05)	.238	-0.07 (-0.20 – 0.05)	.257	-0.07 (-0.20 – 0.06)	.288	-0.09 (-0.22 – 0.04)	.179	-0.08 (-0.21 – 0.05)	.218
Employment status											
	Employed for pay	Ref.	Ref.	Ref.	Ref.	Ref.	Ref.	Ref.	Ref.	Ref.	Ref.
	Unpaid work, incl. housework	0.06 (-0.10 – 0.22)	.435	0.04 (-0.12 – 0.20)	.596	0.06 (-0.10 – 0.23)	.461	0.07 (-0.09 – 0.23)	.397	0.07 (-0.09 – 0.22)	.408
	Unemployed, retired, or disabled	0.03 (-0.08 – 0.14)	.567	0.01 (-0.10 – 0.13)	.819	0.03 (-0.09 – 0.15)	.617	0.04 (-0.08 – 0.15)	.503	0.06 (-0.05 – 0.17)	.276
Monthly personal net income											
	Up to EUR 1,000	Ref.	Ref.	Ref.	Ref.	Ref.	Ref.	Ref.	Ref.	Ref.	Ref.

	EUR 1,001 – EUR 1,500	0.07 (-0.12 – 0.26)	.476	0.09 (-0.10 – 0.29)	.331	0.06 (-0.14 – 0.26)	.554	0.04 (-0.16 – 0.23)	.726	0.03 (-0.16 – 0.22)	.756
	EUR 1,501 – EUR 2,000	0.09 (-0.09 – 0.26)	.325	0.18 (-0.00 – 0.35)	.051	0.16 (-0.03 – 0.34)	.093	0.15 (-0.04 – 0.33)	.116	0.07 (-0.10 – 0.25)	.416
	EUR 2,001 – EUR 2,500	0.08 (-0.10 – 0.25)	.402	0.14 (-0.04 – 0.32)	.122	0.16 (-0.02 – 0.35)	.089	0.16 (-0.02 – 0.35)	.088	0.09 (-0.09 – 0.27)	.331
	EUR 2,501 – EUR 3,000	0.18 (0.00 – 0.35)	.046	0.25 (0.07 – 0.43)	.005	0.27 (0.08 – 0.45)	.004	0.25 (0.07 – 0.44)	.007	0.18 (0.00 – 0.36)	.048
	More than EUR 3,000	0.06 (-0.12 – 0.23)	.545	0.16 (-0.02 – 0.34)	.073	0.22 (0.03 – 0.40)	.020	0.21 (0.03 – 0.39)	.024	0.11 (-0.07 – 0.29)	.248
	No income/NA	-0.04 (-0.25 – 0.18)	.718	0.01 (-0.21 – 0.22)	.948	-0.02 (-0.24 – 0.21)	.887	-0.03 (-0.25 – 0.19)	.797	-0.06 (-0.27 – 0.16)	.611
Educational attainment											
	Low	Ref.	Ref.	Ref.	Ref.	Ref.	Ref.	Ref.	Ref.	Ref.	Ref.
	Medium	0.06 (-0.05 – 0.18)	.288	0.04 (-0.07 – 0.16)	.484	0.05 (-0.07 – 0.18)	.375	0.07 (-0.05 – 0.19)	.271	0.08 (-0.03 – 0.20)	.149
	High	0.08 (-0.04 – 0.20)	.197	0.06 (-0.06 – 0.18)	.298	0.13 (0.00 – 0.25)	.043	0.14 (0.02 – 0.26)	.027	0.15 (0.04 – 0.27)	.010
General privacy concerns											
	Not at all concerned	Ref.	Ref.	Ref.	Ref.						
	Not very concerned	0.07 (-0.07 – 0.21)	.310	0.08 (-0.06 – 0.22)	.275						
	A little concerned	0.14 (-0.00 – 0.27)	.055	0.15 (0.01 – 0.29)	.042						
	Very concerned	-0.05 (-0.24 – 0.14)	.630	-0.04 (-0.23 – 0.16)	.715						
Perceived privacy of information		-0.01 (-0.05 – 0.03)	.599	-0.00 (-0.04 – 0.04)	.990						
Trust in government and research institutions		0.17 (0.10 – 0.23)	<.001	0.18 (0.12 – 0.25)	<.001						
Trust in technology companies		-0.13 (-0.19 – -0.07)	<.001	-0.12 (-0.18 – -0.06)	<.001						
Self-rated health											
	Moderate/Bad	Ref.	Ref.			Ref.	Ref.				
	Good	-0.06 (-0.16 – 0.05)	.320			-0.05 (-0.16 – 0.07)	.438				
	Excellent/Very good	-0.03 (-0.17 – 0.11)	.692			-0.04 (-0.19 – 0.11)	.606				
Chronic illness											
	No	Ref.	Ref.			Ref.	Ref.				
	Yes	0.03 (-0.06 – 0.11)	.543			0.06 (-0.03 – 0.15)	.211				
BMI											
	Underweight/Healthy weight	Ref.	Ref.			Ref.	Ref.				
	Overweight	-0.09 (-0.17 – -0.01)	.031			-0.05 (-0.14 – 0.03)	.237				
	Obesity	-0.03 (-0.13 – 0.07)	.560			-0.03 (-0.14 – 0.08)	.609				

Limited in activities by health	-0.05 (-0.11 – -0.00)	.046			-0.07 (-0.12 – -0.01)	.019				
Difficulties with tasks	0.04 (-0.07 – 0.15)	.455			-0.01 (-0.12 – 0.10)	.889				
No. days with moderate physical activity	-0.01 (-0.02 – 0.01)	.453					-0.00 (-0.02 – 0.02)	.832		
No. days with strenuous physical activity	-0.01 (-0.03 – 0.01)	.241					-0.01 (-0.03 – 0.01)	.276		
No. days walking	-0.00 (-0.02 – 0.02)	.924					0.00 (-0.01 – 0.02)	.639		
No. days running	-0.01 (-0.07 – 0.05)	.664					-0.01 (-0.07 – 0.06)	.876		
No. days biking	-0.00 (-0.02 – 0.02)	.884					-0.00 (-0.02 – 0.02)	.859		
Time sedentary in h	0.01 (-0.01 – 0.02)	.333					0.01 (-0.01 – 0.02)	.283		
Spending time outdoors yesterday										
No	Ref.	Ref.					Ref.	Ref.		
Yes	0.06 (-0.06 – 0.18)	.293					0.09 (-0.04 – 0.21)	.180		
No. of smartphone activities	0.02 (0.00 – 0.03)	.014							0.02 (0.00 – 0.03)	.018
Type of data requested										
Apple Health	Ref.	Ref.							Ref.	Ref.
Google Location History	-0.24 (-0.32 – -0.17)	<.001							-0.25 (-0.34 – -0.17)	<.001
Samsung Health	-0.22 (-0.34 – -0.10)	<.001							-0.24 (-0.36 – -0.11)	<.001
Data example shown										
No	Ref.	Ref.							Ref.	Ref.
Yes	0.04 (-0.03 – 0.11)	.320							0.03 (-0.04 – 0.10)	.387
n	597[a]		603[b]		597[c]		603[d]		603[e]	
AIC	726.6		756.4		787.3		797.4		750.5	
McFadden Pseudo R^2	.227		.155		.111		.107		.157	

[a]Out of the 606 respondents who reported being willing to donate PA data, 9 had to be dropped from the analysis due to missing data in the covariates.
[b]Out of the 606 respondents who reported being willing to donate PA data, 3 had to be dropped from the analysis due to missing data in the covariates.
[c]Out of the 606 respondents who reported being willing to donate PA data, 9 had to be dropped from the analysis due to missing data in the covariates.
[d]Out of the 606 respondents who reported being willing to donate PA data, 3 had to be dropped from the analysis due to missing data in the covariates.
[e]Out of the 606 respondents who reported being willing to donate PA data, 3 had to be dropped from the analysis due to missing data in the covariates.

Table S4. Comparison of results of full logistic regression model predicting successful data donation conditional on starting the donation process (model 4) with more parsimonious models.

	Full model (M4)		Sociodemo., privacy, & trust		Sociodemo. & health		Sociodemo. & PA		Sociodemo. & others	
Variables	AME (95% CI)	P-val.		P-val.	AME (95% CI)	P-val.	AME (95% CI)	P-val.	AME (95% CI)	P-val.
Gender										
Female	Ref.	Ref.	Ref.	Ref.	Ref.	Ref.	Ref.	Ref.	Ref.	Ref.
Male	0.00 (-0.11 – 0.11)	.984	-0.01 (-0.12 – 0.09)	.798	-0.02 (-0.13 – 0.08)	.674	0.00 (-0.10 – 0.11)	.951	0.02 (-0.09 – 0.12)	.743
Age										
50-54 years	Ref.	Ref.	Ref.	Ref.	Ref.	Ref.	Ref.	Ref.	Ref.	Ref.
55-59 years	-0.01 (-0.14 – 0.12)	.859	-0.00 (-0.13 – 0.12)	.951	-0.03 (-0.16 – 0.10)	.631	-0.01 (-0.13 – 0.12)	.886	-0.02 (-0.14 – 0.11)	.765
60-64 years	0.03 (-0.10 – 0.16)	.668	0.00 (-0.13 – 0.14)	.985	0.01 (-0.11 – 0.14)	.829	-0.01 (-0.14 – 0.12)	.900	0.00 (-0.13 – 0.13)	.970
65-69 years	-0.15 (-0.36 – 0.06)	.153	-0.15 (-0.34 – 0.04)	.121	-0.17 (-0.37 – 0.02)	.080	-0.16 (-0.35 – 0.03)	.102	-0.16 (-0.35 – 0.03)	.099
70-74 years	-0.30 (-0.55 – -0.06)	.015	-0.31 (-0.53 – -0.08)	.007	-0.29 (-0.52 – -0.07)	.011	-0.29 (-0.52 – -0.07)	.011	-0.30 (-0.52 – -0.07)	.011
75-79 years	-0.38 (-0.72 – -0.05)	.025	-0.37 (-0.68 – -0.06)	.018	-0.41 (-0.72 – -0.10)	.009	-0.37 (-0.68 – -0.05)	.021	-0.39 (-0.72 – -0.07)	.016
80 years and older	-0.12 (-0.57 – 0.34)	.612	-0.16 (-0.61 – 0.29)	.481	-0.28 (-0.76 – 0.21)	.265	-0.24 (-0.74 – 0.26)	.341	-0.19 (-0.66 – 0.29)	.444
HH size										
Single-person HH	Ref.	Ref.	Ref.	Ref.	Ref.	Ref.	Ref.	Ref.	Ref.	Ref.
Two-person HH	-0.00 (-0.11 – 0.11)	.973	0.02 (-0.09 – 0.13)	.720	0.01 (-0.10 – 0.12)	.829	0.01 (-0.10 – 0.12)	.895	0.00 (-0.11 – 0.11)	.980
Three- and more person HH	-0.11 (-0.26 – 0.05)	.183	-0.09 (-0.25 – 0.06)	.239	-0.11 (-0.27 – 0.05)	.167	-0.12 (-0.28 – 0.03)	.113	-0.13 (-0.28 – 0.02)	.091
Urbanicity										
Not urban	Ref.	Ref.	Ref.	Ref.	Ref.	Ref.	Ref.	Ref.	Ref.	Ref.
Little urban	-0.04 (-0.19 – 0.12)	.643	-0.03 (-0.18 – 0.12)	.688	-0.06 (-0.20 – 0.09)	.461	-0.05 (-0.20 – 0.09)	.481	-0.05 (-0.20 – 0.09)	.484
Moderately urban	0.04 (-0.11 – 0.18)	.620	0.04 (-0.11 – 0.18)	.613	0.01 (-0.14 – 0.15)	.925	0.04 (-0.10 – 0.18)	.590	0.02 (-0.12 – 0.17)	.744
Strongly urban	0.01 (-0.12 – 0.15)	.833	0.02 (-0.12 – 0.15)	.797	0.01 (-0.12 – 0.14)	.866	0.02 (-0.12 – 0.15)	.800	0.01 (-0.12 – 0.14)	.909
Very strongly urban	-0.14 (-0.32 – 0.03)	.109	-0.16 (-0.33 – 0.01)	.068	-0.14 (-0.31 – 0.03)	.104	-0.15 (-0.32 – 0.02)	.080	-0.14 (-0.30 – 0.03)	.113
Employment status										
Employed for pay	Ref.	Ref.	Ref.	Ref.	Ref.	Ref.	Ref.	Ref.	Ref.	Ref.
Unpaid work, incl. housework	0.10 (-0.11 – 0.30)	.360	0.09 (-0.10 – 0.28)	.352	0.06 (-0.14 – 0.27)	.541	0.07 (-0.13 – 0.27)	.505	0.08 (-0.12 – 0.27)	.426
Unemployed, retired, or disabled	0.04 (-0.12 – 0.21)	.603	0.02 (-0.14 – 0.18)	.809	0.03 (-0.13 – 0.19)	.684	0.01 (-0.15 – 0.17)	.866	0.03 (-0.12 – 0.19)	.679
Monthly personal net income										
Up to EUR 1,000	Ref.	Ref.	Ref.	Ref.	Ref.	Ref.	Ref.	Ref.	Ref.	Ref.

	EUR 1,001 – EUR 1,500	-0.14 (-0.40 – 0.13)	.321	-0.09 (-0.39 – 0.20)	.522	-0.15 (-0.42 – 0.13)	.300	-0.15 (-0.43 – 0.13)	.280	-0.15 (-0.41 – 0.11)	.255
	EUR 1,501 – EUR 2,000	-0.07 (-0.31 – 0.16)	.527	-0.01 (-0.27 – 0.24)	.909	-0.07 (-0.31 – 0.17)	.578	-0.07 (-0.31 – 0.18)	.597	-0.10 (-0.32 – 0.13)	.408
	EUR 2,001 – EUR 2,500	-0.03 (-0.26 – 0.21)	.822	0.05 (-0.21 – 0.31)	.702	-0.01 (-0.25 – 0.24)	.956	0.02 (-0.23 – 0.26)	.895	-0.03 (-0.26 – 0.20)	.802
	EUR 2,501 – EUR 3,000	0.05 (-0.18 – 0.28)	.660	0.12 (-0.12 – 0.37)	.326	0.07 (-0.16 – 0.30)	.556	0.07 (-0.17 – 0.31)	.555	0.04 (-0.18 – 0.26)	.738
	More than EUR 3,000	-0.08 (-0.31 – 0.16)	.519	0.03 (-0.22 – 0.28)	.839	-0.02 (-0.26 – 0.22)	.869	-0.03 (-0.27 – 0.22)	.824	-0.06 (-0.28 – 0.17)	.617
	No income/NA	0.04 (-0.25 – 0.34)	.777	-0.01 (-0.35 – 0.33)	.940	0.06 (-0.25 – 0.38)	.686	0.02 (-0.29 – 0.32)	.915	-0.01 (-0.30 – 0.28)	.959
Educational attainment											
	Low	Ref.	Ref.	Ref.	Ref.	Ref.	Ref.	Ref.	Ref.	Ref.	Ref.
	Medium	0.11 (-0.06 – 0.28)	.223	0.09 (-0.07 – 0.25)	.282	0.07 (-0.10 – 0.24)	.425	0.10 (-0.07 – 0.27)	.243	0.10 (-0.07 – 0.27)	.238
	High	0.11 (-0.06 – 0.29)	.200	0.10 (-0.06 – 0.26)	.210	0.09 (-0.08 – 0.26)	.291	0.12 (-0.04 – 0.29)	.141	0.15 (-0.02 – 0.31)	.082
General privacy concerns											
	Not at all concerned	Ref.	Ref.	Ref.	Ref.						
	Not very concerned	-0.00 (-0.20 – 0.20)	.999	0.01 (-0.18 – 0.21)	.885						
	A little concerned	0.02 (-0.18 – 0.22)	.830	0.04 (-0.15 – 0.24)	.674						
	Very concerned	0.13 (-0.11 – 0.38)	.285	0.15 (-0.09 – 0.39)	.207						
Perceived privacy of information		0.03 (-0.02 – 0.08)	.279	0.03 (-0.02 – 0.08)	.202						
Trust in government and research institutions		0.02 (-0.07 – 0.11)	.622	0.06 (-0.03 – 0.14)	.200						
Trust in technology companies		-0.02 (-0.10 – 0.06)	.565	-0.03 (-0.11 – 0.04)	.392						
Self-rated health											
	Moderate/Bad	Ref.	Ref.			Ref.	Ref.				
	Good	-0.00 (-0.16 – 0.15)	.961			-0.01 (-0.16 – 0.14)	.923				
	Excellent/Very good	0.00 (-0.19 – 0.20)	.960			-0.02 (-0.21 – 0.17)	.812				
Chronic illness											
	No	Ref.	Ref.			Ref.	Ref.				
	Yes	0.01 (-0.10 – 0.12)	.897			0.01 (-0.10 – 0.12)	.918				
BMI											
	Underweight/Healthy weight	Ref.	Ref.			Ref.	Ref.				
	Overweight	-0.01 (-0.11 – 0.10)	.878			0.01 (-0.09 – 0.12)	.812				
	Obesity	-0.08 (-0.23 – 0.07)	.274			-0.08 (-0.22 – 0.07)	.303				

Limited in activities by health	-0.01 (-0.08 – 0.06)	.790			-0.01 (-0.09 – 0.06)	.704				
Difficulties with tasks	-0.08 (-0.23 – 0.08)	.316			-0.09 (-0.24 – 0.06)	.233				
No. days with moderate physical activity	0.00 (-0.02 – 0.02)	.917					-0.01 (-0.03 – 0.02)	.655		
No. days with strenuous physical activity	0.01 (-0.02 – 0.04)	.595					0.01 (-0.01 – 0.04)	.325		
No. days walking	-0.01 (-0.04 – 0.01)	.241					-0.00 (-0.02 – 0.02)	.845		
No. days running	-0.06 (-0.14 – 0.03)	.174					-0.04 (-0.12 – 0.04)	.299		
No. days biking	0.00 (-0.02 – 0.03)	.772					0.00 (-0.02 – 0.03)	.881		
Time sedentary in h	0.01 (-0.00 – 0.03)	.117					0.01 (-0.00 – 0.03)	.074		
Spending time outdoors yesterday										
No	Ref.	Ref.					Ref.	Ref.		
Yes	0.04 (-0.13 – 0.22)	.623					0.05 (-0.12 – 0.23)	.574		
No. of smartphone activities	0.01 (-0.01 – 0.03)	.206							0.01 (-0.01 – 0.03)	.280
Type of data requested										
Apple Health	Ref.	Ref.							Ref.	Ref.
Google Location History	-0.10 (-0.21 – 0.00)	.058							-0.10 (-0.21 – 0.00)	.055
Samsung Health	-0.02 (-0.18 – 0.14)	.796							-0.05 (-0.21 – 0.11)	.524
Data example shown										
No	Ref.	Ref.							Ref.	Ref.
Yes	0.02 (-0.07 – 0.11)	.629							0.02 (-0.07 – 0.11)	.698
n	351[a]		354		351[b]		354		354	
AIC	452.8		434.7		434.0		438.3		431.7	
McFadden Pseudo R^2	.138		.102		.101		.099		.101	

[a]Out of the 354 respondents who started to donate PA data, 3 had to be dropped from the analysis due to missing data in the covariates.

[b]Out of the 354 respondents who started to donate PA data, 3 had to be dropped from the analysis due to missing data in the covariates.
