# Supplementary material for: Data Donation as a Method to Measure Physical Activity in Older Adults: Cross-Sectional Web Survey Assessing Consent Rates, Donation Success, and Bias
Source: J Med Internet Res. 2025 Sep 26;27:e69799. doi: 10.2196/69799 (PMC12514404; doi:10.2196/69799)
Supplement: Multimedia Appendix 5 [file jmir_v27i1e69799_app5.pdf]

Multimedia Appendix 5

Table S1. Average marginal effects (AME), 95% confidence intervals (CI), and *P* values from logistic regression model predicting owning an iPhone or Android phone (model 1).

Variables		AME (95% CI)	*P* value
**Gender**			
	Female	Ref.	Ref.
	Male	-0.05 (-0.08 – -0.02)	.001
**Age**			
	50-54 years	Ref.	Ref.
	55-59 years	-0.01 (-0.05 – 0.03)	.649
	60-64 years	-0.02 (-0.06 – 0.02)	.287
	65-69 years	-0.03 (-0.07 – 0.02)	.218
	70-74 years	-0.05 (-0.09 – 0.00)	.063
	75-79 years	-0.08 (-0.14 – -0.03)	.004
	80 years and older	-0.19 (-0.27 – -0.12)	<.001
**HH size**			
	Single-person HH	Ref.	Ref.
	Two-person HH	0.03 (-0.00 – 0.06)	.101
	Three- and more person HH	0.01 (-0.04 – 0.06)	.594
**Urbanicity**			
	Not urban	Ref.	Ref.
	Little urban	0.02 (-0.02 – 0.06)	.371
	Moderately urban	0.01 (-0.03 – 0.06)	.582
	Strongly urban	0.01 (-0.03 – 0.05)	.524
	Very strongly urban	0.01 (-0.04 – 0.06)	.667
**Employment status**			
	Employed for pay	Ref.	Ref.
	Unpaid work, incl. housework	-0.02 (-0.07 – 0.04)	.533
	Unemployed, retired, or disabled	-0.00 (-0.05 – 0.04)	.932
**Monthly personal net income**			
	Up to EUR 1,000	Ref.	Ref.
	EUR 1,001 – EUR 1,500	0.04 (-0.02 – 0.10)	.164
	EUR 1,501 – EUR 2,000	0.07 (0.02 – 0.13)	.012
	EUR 2,001 – EUR 2,500	0.08 (0.03 – 0.14)	.005
	EUR 2,501 – EUR 3,000	0.06 (0.00 – 0.13)	.046
	More than EUR 3,000	0.11 (0.05 – 0.17)	<.001
	No income/NA	0.00 (-0.07 – 0.07)	.994
**Educational attainment**			
	Low	Ref.	Ref.
	Medium	0.03 (-0.00 – 0.07)	.061
	High	0.06 (0.03 – 0.10)	<.001
**General privacy concerns**			
	Not at all concerned	Ref.	Ref.
	Not very concerned	0.06 (0.01 – 0.11)	.027
	A little concerned	0.08 (0.03 – 0.14)	.001
	Very concerned	0.05 (-0.01 – 0.12)	.121
**Perceived privacy of information**		0.00 (-0.01 – 0.02)	.503

**Trust in government and research institutions**		0.00 (-0.02 – 0.02)	.901
**Trust in technology companies**		0.01 (-0.01 – 0.03)	.282
**Self-rated health**			
	Moderate/Bad	Ref.	Ref.
	Good	-0.01 (-0.04 – 0.02)	.544
	Excellent/Very good	-0.01 (-0.06 – 0.04)	.792
**Chronic illness**			
	No	Ref.	Ref.
	Yes	-0.02 (-0.05 – 0.01)	.134
**BMI**			
	Underweight/Healthy weight	Ref.	Ref.
	Overweight	0.01 (-0.01 – 0.04)	.329
	Obesity	0.00 (-0.03 – 0.04)	.811
**Limited in activities by health**		0.01 (-0.01 – 0.02)	.349
**Difficulties with tasks**		-0.05 (-0.07 – -0.02)	<.001
**No. days with moderate physical activity**		0.00 (-0.00 – 0.01)	.291
**No. days with strenuous physical activity**		0.00 (-0.00 – 0.01)	.510
**No. days walking**		0.00 (-0.00 – 0.01)	.187
**No. days running**		-0.01 (-0.03 – 0.01)	.322
**No. days biking**		-0.01 (-0.01 – -0.00)	.001
**Time sedentary in h**		0.00 (-0.00 – 0.00)	.865
**Spending time outdoors yesterday**			
	No	Ref.	Ref.
	Yes	-0.01 (-0.04 – 0.02)	.587
n		2,020a	
AIC		1,117.8	
McFadden Pseudo R^2		.174	

[a]Out of the 2,086 respondents in the survey, 66 had to be dropped from the analysis due to missing data in the covariates.

Table S2. Average marginal effects (AME), 95% confidence intervals (CI), and *P* values from logistic regression model predicting willingness to donate PA data conditional on being asked (model 2).

Variables	AME (95% CI)	*P* value
**Gender**		
Female	Ref.	Ref.
Male	0.07 (0.02 – 0.11)	.006
**Age**		
50-54 years	Ref.	Ref.
55-59 years	0.00 (-0.07 – 0.08)	.967
60-64 years	-0.07 (-0.15 – 0.01)	.087
65-69 years	-0.09 (-0.18 – 0.00)	.051
70-74 years	-0.08 (-0.18 – 0.02)	.097
75-79 years	-0.14 (-0.24 – -0.03)	.011
80 years and older	-0.14 (-0.26 – -0.02)	.020
**HH size**		
Single-person HH	Ref.	Ref.
Two-person HH	-0.07 (-0.12 – -0.01)	.015
Three- and more person HH	-0.10 (-0.16 – -0.03)	.004
**Urbanicity**		
Not urban	Ref.	Ref.
Little urban	-0.04 (-0.12 – 0.03)	.245
Moderately urban	-0.08 (-0.15 – -0.00)	.036
Strongly urban	-0.11 (-0.18 – -0.05)	.001
Very strongly urban	-0.10 (-0.18 – -0.03)	.006
**Employment status**		
Employed for pay	Ref.	Ref.
Unpaid work, incl. housework	-0.02 (-0.10 – 0.07)	.692
Unemployed, retired, or disabled	0.03 (-0.03 – 0.10)	.315
**Monthly personal net income**		
Up to EUR 1,000	Ref.	Ref.
EUR 1,001 – EUR 1,500	-0.01 (-0.10 – 0.08)	.834
EUR 1,501 – EUR 2,000	0.02 (-0.07 – 0.11)	.718
EUR 2,001 – EUR 2,500	-0.02 (-0.11 – 0.07)	.684
EUR 2,501 – EUR 3,000	0.03 (-0.06 – 0.13)	.479
More than EUR 3,000	0.05 (-0.05 – 0.15)	.313
No income/NA	-0.07 (-0.17 – 0.03)	.154
**Educational attainment**		
Low	Ref.	Ref.
Medium	0.05 (-0.00 – 0.11)	.057
High	0.10 (0.04 – 0.16)	.001
**General privacy concerns**		
Not at all concerned	Ref.	Ref.
Not very concerned	-0.03 (-0.11 – 0.06)	.503
A little concerned	-0.06 (-0.14 – 0.03)	.186
Very concerned	-0.02 (-0.13 – 0.09)	.711
**Perceived privacy of information**	-0.04 (-0.06 – -0.02)	<.001
**Trust in government and research institutions**	0.05 (0.01 – 0.09)	.006
**Trust in technology companies**	0.01 (-0.02 – 0.05)	.530

**Self-rated health**			
	Moderate/Bad	Ref.	Ref.
	Good	-0.06 (-0.13 – 0.00)	.056
	Excellent/Very good	-0.05 (-0.13 – 0.03)	.251
**Chronic illness**			
	No	Ref.	Ref.
	Yes	0.06 (0.01 – 0.11)	.011
**BMI**			
	Underweight/Healthy weight	Ref.	Ref.
	Overweight	-0.04 (-0.08 – 0.01)	.105
	Obesity	-0.02 (-0.08 – 0.04)	.421
**Limited in activities by health**		-0.02 (-0.05 – 0.01)	.155
**Difficulties with tasks**		0.00 (-0.06 – 0.06)	.980
**No. days with moderate physical activity**		-0.00 (-0.01 – 0.01)	.956
**No. days with strenuous physical activity**		0.01 (-0.00 – 0.02)	.181
**No. days walking**		-0.00 (-0.01 – 0.01)	.592
**No. days running**		-0.02 (-0.05 – 0.02)	.271
**No. days biking**		0.01 (-0.00 – 0.02)	.231
**Time sedentary in h**		0.00 (-0.00 – 0.01)	.278
**Spending time outdoors yesterday**			
	No	Ref.	Ref.
	Yes	0.04 (-0.02 – 0.10)	.157
**No. of smartphone activities**		0.03 (0.02 – 0.04)	<.001
**Type of data requested**			
	Apple Health	Ref.	Ref.
	Google Location History	-0.14 (-0.18 – -0.09)	<.001
	Samsung Health	-0.07 (-0.14 – 0.00)	.061
**Data example shown**			
	No	Ref.	Ref.
	Yes	-0.05 (-0.09 – -0.01)	.023
n		1,830[a]	
AIC		2,097.5	
McFadden Pseudo R^2		.134	

[a]Out of the 1,883 respondents who were asked the willingness question, 53 had to be dropped from the analysis due to missing data in the covariates.

Table S3. Fine-grained coding of answers to open-ended question on reasons for not being willing to donate PA data.

Code		Reason	n (%)[a]
**1**		**Need to protect privacy**	269 (21.3%)
	1.1	Generally not willing to provide data	256 (20.3%)
	1.2	Already too much data shared	16 (1.3%)
**2**		**Concern about data misuse/data breach**	44 (3.5%)
	2.1	General data protection concerns	31 (2.5%)
	2.2	Missing information on how data will be protected	4 (0.3%)
	2.3	Worries about third party access to data	10 (0.8%)
**3**		**Anticipating difficulties with data donation**	339 (26.8%)
	3.1	Anticipated technical problems	38 (3%)
	3.2	Too complicated	79 (6.3%)
	3.3	Too cumbersome	194 (15.4%)
	3.4	Other practical issues	47 (3.7%)
**4**		**Perceived usefulness**	257 (20.3%)
4.1		Too few data are generated	135 (10.7%)
4.2		Study goal seems not useful	12 (1%)
4.3		Usefulness generally doubted without specification	67 (5.3%)
4.4		Not right person for the study	113 (8.9%)
**5**		**Personal reasons**	106 (8.4%)
	5.1	Concern about some personal disadvantages	8 (0.6%)
	5.2	Health issues make data donation impossible	70 (5.5%)
	5.3	Current living conditions make data donation impossible	17 (1.3%)
	5.4	No personal benefit/No interest in data	11 (0.9%)
**6**		**Other reasons**	96 (7.6%)
	6.1	No trust in researchers/university	26 (2.1%)
	6.2	Not enough information	12 (1%)
	6.3	Incentive too small	15 (1.2%)
	6.4	Concerns related to firms that collect data (Apple, Google, Samsung)	26 (2.1%)
	6.5	LISS-related reasons	18 (1.4%)
**7**		**Unconcrete refusal**	238 (18.8%)
	7.1	Don't want to	223 (17.7%)
	7.2	General concern/Uncertainty	16 (1.3%)
**8**		**No reasons provided**	100 (7.9%)
	8.1	No reason/DK	60 (4.8%)
	8.2	No useful/sensical response	33 (2.6%)
	8.3	Unrelated comment	7 (0.6%)

Notes: n=1,263

[a]Each answer could be coded in multiple categories, thus percentages can add up to more than 100.

Table S4. Average marginal effects (AME), 95% confidence intervals (CI), and *P* values from logistic regression model predicting starting to donate PA data conditional on willingness (model 3).

Variables		AME (95% CI)	*P* value
**Gender**			
	Female	Ref.	Ref.
	Male	0.04 (-0.04 – 0.12)	.315
**Age**			
	50-54 years	Ref.	Ref.
	55-59 years	-0.08 (-0.19 – 0.03)	.161
	60-64 years	-0.10 (-0.22 – 0.02)	.098
	65-69 years	-0.20 (-0.34 – -0.06)	.005
	70-74 years	-0.19 (-0.35 – -0.04)	.015
	75-79 years	-0.41 (-0.59 – -0.24)	<.001
	80 years and older	-0.44 (-0.65 – -0.23)	<.001
**HH size**			
	Single-person HH	Ref.	Ref.
	Two-person HH	0.06 (-0.03 – 0.15)	.173
	Three- and more person HH	-0.04 (-0.15 – 0.08)	.520
**Urbanicity**			
	Not urban	Ref.	Ref.
	Little urban	-0.01 (-0.13 – 0.10)	.808
	Moderately urban	0.00 (-0.11 – 0.12)	.956
	Strongly urban	-0.03 (-0.14 – 0.08)	.578
	Very strongly urban	-0.07 (-0.20 – 0.05)	.238
**Employment status**			
	Employed for pay	Ref.	Ref.
	Unpaid work, incl. housework	0.06 (-0.10 – 0.22)	.435
	Unemployed, retired, or disabled	0.03 (-0.08 – 0.14)	.567
**Monthly personal net income**			
	Up to EUR 1,000	Ref.	Ref.
	EUR 1,001 – EUR 1,500	0.07 (-0.12 – 0.26)	.476
	EUR 1,501 – EUR 2,000	0.09 (-0.09 – 0.26)	.325
	EUR 2,001 – EUR 2,500	0.08 (-0.10 – 0.25)	.402
	EUR 2,501 – EUR 3,000	0.18 (0.00 – 0.35)	.046
	More than EUR 3,000	0.06 (-0.12 – 0.23)	.545
	No income/NA	-0.04 (-0.25 – 0.18)	.718
**Educational attainment**			
	Low	Ref.	Ref.
	Medium	0.06 (-0.05 – 0.18)	.288
	High	0.08 (-0.04 – 0.20)	.197
**General privacy concerns**			
	Not at all concerned	Ref.	Ref.
	Not very concerned	0.07 (-0.07 – 0.21)	.310
	A little concerned	0.14 (-0.00 – 0.27)	.055
	Very concerned	-0.05 (-0.24 – 0.14)	.630
**Perceived privacy of information**		-0.01 (-0.05 – 0.03)	.599
**Trust in government and research institutions**		0.17 (0.10 – 0.23)	<.001
**Trust in technology companies**		-0.13 (-0.19 – -0.07)	<.001

**Self-rated health**			
	Moderate/Bad	Ref.	Ref.
	Good	-0.06 (-0.16 – 0.05)	.320
	Excellent/Very good	-0.03 (-0.17 – 0.11)	.692
**Chronic illness**			
	No	Ref.	Ref.
	Yes	0.03 (-0.06 – 0.11)	.543
**BMI**			
	Underweight/Healthy weight	Ref.	Ref.
	Overweight	-0.09 (-0.17 – -0.01)	.031
	Obesity	-0.03 (-0.13 – 0.07)	.560
**Limited in activities by health**		-0.05 (-0.11 – -0.00)	.046
**Difficulties with tasks**		0.04 (-0.07 – 0.15)	.455
**No. days with moderate physical activity**		-0.01 (-0.02 – 0.01)	.453
**No. days with strenuous physical activity**		-0.01 (-0.03 – 0.01)	.241
**No. days walking**		-0.00 (-0.02 – 0.02)	.924
**No. days running**		-0.01 (-0.07 – 0.05)	.664
**No. days biking**		-0.00 (-0.02 – 0.02)	.884
**Time sedentary in h**		0.01 (-0.01 – 0.02)	.333
**Spending time outdoors yesterday**			
	No	Ref.	Ref.
	Yes	0.06 (-0.06 – 0.18)	.293
**No. of smartphone activities**		0.02 (0.00 – 0.03)	.014
**Type of data requested**			
	Apple Health	Ref.	Ref.
	Google Location History	-0.24 (-0.32 – -0.17)	<.001
	Samsung Health	-0.22 (-0.34 – -0.10)	<.001
**Data example shown**			
	No	Ref.	Ref.
	Yes	0.04 (-0.03 – 0.11)	.320
n		597[a]	
AIC		726.6	
McFadden Pseudo R^2		.227	

[a]Out of the 606 respondents who reported being willing to donate PA data, 9 had to be dropped from the analysis due to missing data in the covariates.

Table S5. Average marginal effects (AME), 95% confidence intervals (CI), and *P* values from logistic regression model predicting successful data donation conditional on starting the donation process (model 4).

Variables		AME (95% CI)	*P* value
**Gender**			
	Female	Ref.	Ref.
	Male	0.00 (-0.11 – 0.11)	.984
**Age**			
	50-54 years	Ref.	Ref.
	55-59 years	-0.01 (-0.14 – 0.12)	.859
	60-64 years	0.03 (-0.10 – 0.16)	.668
	65-69 years	-0.15 (-0.36 – 0.06)	.153
	70-74 years	-0.30 (-0.55 – -0.06)	.015
	75-79 years	-0.38 (-0.72 – -0.05)	.025
	80 years and older	-0.12 (-0.57 – 0.34)	.612
**HH size**			
	Single-person HH	Ref.	Ref.
	Two-person HH	-0.00 (-0.11 – 0.11)	.973
	Three- and more person HH	-0.11 (-0.26 – 0.05)	.183
**Urbanicity**			
	Not urban	Ref.	Ref.
	Little urban	-0.04 (-0.19 – 0.12)	.643
	Moderately urban	0.04 (-0.11 – 0.18)	.620
	Strongly urban	0.01 (-0.12 – 0.15)	.833
	Very strongly urban	-0.14 (-0.32 – 0.03)	.109
**Employment status**			
	Employed for pay	Ref.	Ref.
	Unpaid work, incl. housework	0.10 (-0.11 – 0.30)	.360
	Unemployed, retired, or disabled	0.04 (-0.12 – 0.21)	.603
**Monthly personal net income**			
	Up to EUR 1,000	Ref.	Ref.
	EUR 1,001 – EUR 1,500	-0.14 (-0.40 – 0.13)	.321
	EUR 1,501 – EUR 2,000	-0.07 (-0.31 – 0.16)	.527
	EUR 2,001 – EUR 2,500	-0.03 (-0.26 – 0.21)	.822
	EUR 2,501 – EUR 3,000	0.05 (-0.18 – 0.28)	.660
	More than EUR 3,000	-0.08 (-0.31 – 0.16)	.519
	No income/NA	0.04 (-0.25 – 0.34)	.777
**Educational attainment**			
	Low	Ref.	Ref.
	Medium	0.11 (-0.06 – 0.28)	.223
	High	0.11 (-0.06 – 0.29)	.200
**General privacy concerns**			
	Not at all concerned	Ref.	Ref.
	Not very concerned	-0.00 (-0.20 – 0.20)	.999
	A little concerned	0.02 (-0.18 – 0.22)	.830
	Very concerned	0.13 (-0.11 – 0.38)	.285
**Perceived privacy of information**		0.03 (-0.02 – 0.08)	.279
**Trust in government and research institutions**		0.02 (-0.07 – 0.11)	.622
**Trust in technology companies**		-0.02 (-0.10 – 0.06)	.565

**Self-rated health**			
	Moderate/Bad	Ref.	Ref.
	Good	-0.00 (-0.16 – 0.15)	.961
	Excellent/Very good	0.00 (-0.19 – 0.20)	.960
**Chronic illness**			
	No	Ref.	Ref.
	Yes	0.01 (-0.10 – 0.12)	.897
**BMI**			
	Underweight/Healthy weight	Ref.	Ref.
	Overweight	-0.01 (-0.11 – 0.10)	.878
	Obesity	-0.08 (-0.23 – 0.07)	.274
**Limited in activities by health**		-0.01 (-0.08 – 0.06)	.790
**Difficulties with tasks**		-0.08 (-0.23 – 0.08)	.316
**No. days with moderate physical activity**		0.00 (-0.02 – 0.02)	.917
**No. days with strenuous physical activity**		0.01 (-0.02 – 0.04)	.595
**No. days walking**		-0.01 (-0.04 – 0.01)	.241
**No. days running**		-0.06 (-0.14 – 0.03)	.174
**No. days biking**		0.00 (-0.02 – 0.03)	.772
**Time sedentary in h**		0.01 (-0.00 – 0.03)	.117
**Spending time outdoors yesterday**			
	No	Ref.	Ref.
	Yes	0.04 (-0.13 – 0.22)	.623
**No. of smartphone activities**		0.01 (-0.01 – 0.03)	.206
**Type of data requested**			
	Apple Health	Ref.	Ref.
	Google Location History	-0.10 (-0.21 – 0.00)	.058
	Samsung Health	-0.02 (-0.18 – 0.14)	.796
**Data example shown**			
	No	Ref.	Ref.
	Yes	0.02 (-0.07 – 0.11)	.629
n		351[a]	
AIC		452.8	
McFadden Pseudo R^2		.138	

[a]Out of the 354 respondents who started to donate PA data, 3 had to be dropped from the analysis due to missing data in the covariates.

Table S6. Bias (in percentage points) and 95% confidence intervals (CI) between donors and the full sample.

Variables		Bias (percentage points)	95% CI
**Gender**			
	Female	-6.7	-12.5 – -0.9
	Male	+6.7	1.2 – 12.3
**Age**			
	50-54 years	+11.4	7.1 – 15.7
	55-59 years	+7.7	3.4 – 12.0
	60-64 years	+4.2	-0.3 – 8.8
	65-69 years	-0.2	-4.5 – 4.0
	70-74 years	-5.0	-8.9 – -1.1
	75-79 years	-10.0	-12.5 – -7.5
	80 years and older	-8.0	-9.9 – -6.1
**HH size**			
	Single-person HH	-4.0	-8.8 – 0.9
	Two-person HH	-1.4	-7.2 – 4.3
	Three- and more person HH	+5.4	0.7 – 10.1
**Urbanicity**			
	Not urban	+4.0	-0.2 – 8.2
	Little urban	+0.1	-4.3 – 4.5
	Moderately urban	-0.1	-4.7 – 4.6
	Strongly urban	+0.2	-5.0 – 5.4
	Very strongly urban	-4.2	-8.3 – 0.0
**Employment status**			
	Employed for pay	+22.9	17.8 – 28.0
	Unpaid work, incl. housework	-5.3	-8.5 – -2.2
	Unemployed, retired, or disabled	-17.5	-23.2 – -11.8
**Monthly personal net income**			
	Up to EUR 1,000	-6.5	-9.2 – -3.7
	EUR 1,001 – EUR 1,500	-8.6	-11.8 – -5.4
	EUR 1,501 – EUR 2,000	-3.8	-7.8 – 0.2
	EUR 2,001 – EUR 2,500	+0.8	-3.4 – 5.0
	EUR 2,501 – EUR 3,000	+6.7	2.6 – 10.9
	More than EUR 3,000	+15.5	10.7 – 20.3
	No income/NA	-4.2	-7.0 – -1.4
**Educational attainment**			
	Low	-17.5	-21.5 – -13.5
	Medium	+0.3	-5.1 – 5.8
	High	+17.4	12.0 – 22.7
**General privacy concerns**			
	Not at all concerned	-2.5	-5.4 – 0.4
	Not very concerned	+1.0	-4.4 – 6.5
	A little concerned	+4.4	-1.2 – 10.1
	Very concerned	-2.9	-5.9 – 0.1
**Perceived privacy of personal information**			
	Low	-2.5	-7.8 – 2.9
	Medium	+3.0	-2.4 – 8.4
	High	-0.5	-5.9 – 4.9

**Trust in government and research organizations**			
	Low	-13.4	-18.4 – -8.4
	Medium	-3.6	-9.0 – 1.7
	High	+17.1	11.8 – 22.4
**Trust in technology companies**			
	Low	+5.0	-0.5 – 10.4
	Medium	-5.6	-10.9 – -0.3
	High	+0.7	-4.7 – 6.1
**Self-rated health**			
	Moderate/Bad	-5.0	-9.7 – -0.2
	Good	-1.4	-7.1 – 4.3
	Excellent/Very good	+6.3	1.7 – 10.9
**Chronic illness**			
	No	1.1	-4.5 – 6.8
	Yes	-2.6	-8.2 – 3.0
**BMI**			
	Underweight/Healthy weight	+2.2	-3.5 – 7.9
	Overweight	+1.4	-4.2 – 7.0
	Obesity	-3.0	-7.2 – 1.1
**Limited activities by health**			
	Low	+8.5	3.1 – 13.9
	Medium	+2.5	-3.0 – 7.9
	High	-11.0	-16.0 – -5.9
**Difficulties with tasks**			
	Low	+10.0	4.6 – 15.4
	Medium	+3.6	-1.8 – 9.1
	High	-13.7	-18.6 – -8.7
**No. days with moderate physical activity**			
	Low	-5.9	-11.2 – -0.6
	Medium	+6.5	1.0 – 11.9
	High	-0.6	-6.0 – 4.9
**No. days with strenuous physical activity**			
	Low	-9.6	-14.7 – -4.4
	Medium	+5.0	-0.5 – 10.4
	High	+4.6	-0.8 – 10.1
**No. days walking**			
	Low	-7.6	-12.8 – -2.3
	Medium	+3.8	-1.6 – 9.2
	High	+3.8	-1.6 – 9.2
**No. days running**			
	Low	-1.7	-7.1 – 3.7
	Medium	-0.1	-5.5 – 5.3
	High	+1.8	-3.6 – 7.3
**No. days biking**			
	Low	-6.8	-12.1 – -1.5
	Medium	+3.7	-1.7 – 9.2
	High	+3.1	-2.4 – 8.5
**Time sedentary in h**			
	Low	-5.2	-10.5 – 0.1

	Medium	-3.2	-8.6 – 2.1
	High	+8.5	3.1 – 13.9
**Spending time outdoors yesterday**			
	No	-6.2	-9.8 – -2.5
	Yes	+6.2	2.7 – 9.6
